# Promoting collective cooperation through temporal interactions

**DOI:** 10.1073/pnas.2509575122

**Published:** 2025-06-27

**Authors:** Yao Meng, Alex McAvoy, Aming Li

**Affiliations:** ^a^Center for Systems and Control, College of Engineering, Peking University, Beijing 100871, China; ^b^School of Data Science and Society, University of North Carolina at Chapel Hill, Chapel Hill, NC 27599; ^c^Department of Mathematics, University of North Carolina at Chapel Hill, Chapel Hill, NC 27599; ^d^Center for Multi-Agent Research, Institute for Artificial Intelligence, Peking University, Beijing 100871, China

**Keywords:** temporal networks, collective cooperation, evolutionary game dynamics

## Abstract

Collective cooperation maintains the function of many natural and social systems, making understanding the evolution of cooperation a central question of modern science. Although human interactions involve complex contact networks, current explorations are limited to static networks, where social ties are permanent and do not change over time. In reality, human activities often involve temporal interactions, where links are impermanent, and understanding the evolution of cooperation on such temporal networks is an open problem. Here, we systematically analyze how cooperation spreads on arbitrary temporal networks, and we distill our results down to a concise condition, which integrates evolutionary game dynamics with both static and temporal interactions. We find that the emergence of cooperation is facilitated by a simple rule of thumb: Hubs (individuals with many social ties) should be temporally deprioritized in interactions. For empirical applications, we further provide a quantitative metric capturing the priority of hubs, which is validated on empirical datasets based on its effectiveness in orchestrating the ordering of interactions to best promote cooperation. Our findings unveil the fundamental advantages conferred by temporal interactions for promoting collective cooperation, transcending the specific insights gleaned from studying static networks.

## Significance

Human interpersonal interactions are the building blocks of widespread collective cooperative behaviors, and how cooperation can emerge in interactions between selfish individuals is important to understand. Traditionally, static networks, wherein links are permanent, have been the most common framework for studying this open problem. However, in many natural and social systems, links are intermittent and the contact structure is better described using temporal networks. Here, we offer an analytical condition under which cooperation can emerge with arbitrary temporal interactions. To best promote cooperation, we find that hubs with many social links should be involved in later interactions, and we develop an efficient metric for designing temporal contact structures in empirical networks.

Explaining the prevalence of altruistic behaviors among self-interested individuals has been a central topic in modern science (1–6), dating back to the seminal work of Hamilton (7). In recent decades, researchers have sought to explain the emergence of cooperation in finely structured populations using evolutionary game theory (3, 8–11), where the extensive variety of interpersonal interactions that humans engage in on a daily basis can be modeled using complex networks (4, 12–19). A major strand of ongoing research concerns understanding how cooperation self-organizes through a combination of network structure and the mechanism of behavioral transmission as well as how empirical data on these two factors can be incorporated into collective population dynamics (17, 20–25).

Although existing research has elucidated the role of network structure in the spread of social traits, many such studies rely on the key assumption that the underlying network structure is static, where interactions represented by network links are permanent and do not change over time. Within this paradigm, heterogeneous structures—in which individuals can have different numbers of neighbors—have long been recognized as important for capturing realistic populations in modeling approaches (17, 26, 27). Qualitatively novel dynamics and properties emerge in empirical systems modeled by heterogeneous networks, which are absent from homogeneous topologies wherein all individuals have the same number of neighbors (8, 28). For example, in public goods games, heterogeneous networks can lead to heightened wealth inequality, following a power-law distribution on scale-free networks (27). Such networks can also promote the spread of inefficient prosocial behaviors, in which the costs vastly exceed the benefits (29).

In reality, static heterogeneous networks involve the aggregation of dynamic interactions over time, which inevitably results in the loss of information about when interactions occur (30). They also fall short of capturing the intrinsic temporal characteristics of human social interactions. Indeed, numerous interpersonal exchanges are dynamic and impermanent, characterized by networks that vary over time (31–36). Instances include electronic communication through both email and question-and-answer websites (e.g., Stack Overflow) (37), as well as face-to-face interactions like those in workplaces (38) and schools (39). Although precise timestamped data on temporal networks are not always readily available, randomness in interaction patterns across species (e.g., due to weather, seasonality, travel, or migration) suggests that temporally varying contact networks are integral components of social evolution, and understanding the emergence of cooperation on such ubiquitous temporal networks remains an open problem.

Here, we derive a mathematical condition for when cooperation emerges on temporal networks, which includes an explicit description of how network transitions are incorporated. Our findings reveal that prioritizing individuals with fewer social ties is crucial for the efficient spread of cooperative behaviors over time. As applications, we design optimal temporal orderings of interactions on both synthetic and empirical datasets. Our theoretical results are general and are developed with future applications in mind, as more empirical data about temporal interactions become available.

## Results

**Fig. 1.**
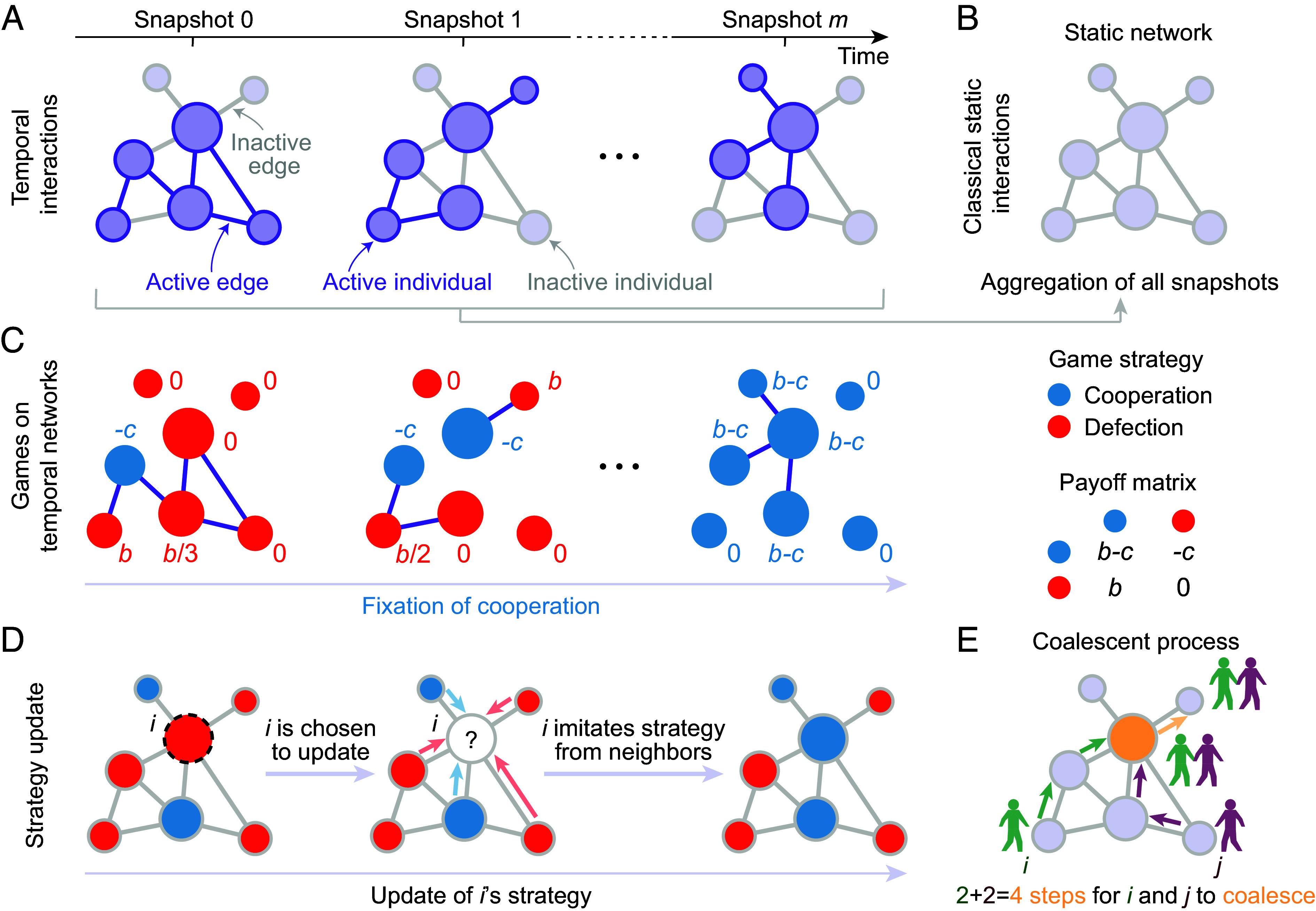
Evolutionary games with temporal interactions. (*A*) Temporal interactions between individuals over time are represented by a snapshot sequence, where the active edges at each time step are marked in dark purple, and an individual is active if it has at least one active edge. Edges or nodes that appear in the static network but are inactive in the snapshots are marked in gray. (*B*) The static network aggregated from all snapshots in the temporal network captures the social relationships among individuals. (*C*) Individuals choose either cooperation (blue) or defection (red) as their strategy in the game. At each time step, each individual, *i*, plays the donation game with all current neighbors and obtains an average payoff, *f*_*i*_, where a cooperator pays a cost *c* to provide a benefit *b* to its opponent, and defectors provide no benefit and pay no cost. (*D*) After each time step of interactions, a randomly selected individual, *i* (dashed circle), updates its strategy following an imitation mechanism (13, 17). The probability that *i* copies the behavior of neighbor *j* is proportional to *j*’s fitness, $F_{j}=1+\delta f_{j}$. Starting from a single cooperator, the evolutionary process ends when cooperators either fix or go extinct. (*E*) Random walkers starting from *i* and *j* meet on the yellow node and then continue to make a single random walk, in this example coalescing in four time steps, with each taking two steps before they meet.

We consider games with temporal interactions in a population of *N* individuals, where the network structures can vary with time (Fig. 1*A*) By combining all interaction snapshots over time, the social relationships are represented by an aggregated static network (Fig. 1*B*). This structure is also known as the replacement network (17, 40, 41), which captures who can imitate whom during the evolutionary process (Fig. 1*B*).

Individuals choose either cooperation or defection. At each time step, each individual, *i*, plays the game pairwise with its interaction partners and obtains an average payoff, *f*_*i*_. A cooperator pays a cost, *c*, to provide a benefit, *b*, to its opponent. Defectors pay nothing and provide no benefit (Fig. 1*C*). An individual, *i*, is then chosen uniformly at random from the population to imitate a strategy from one of its neighbors, *j*, with probability proportional to the fitness of *j* (*Materials and Methods*). This commonly used “fitness” (9, 13, 17) is defined as $F_{j}=1+\delta f_{j}$, where *δ* > 0 captures the intensity of selection (Fig. 1*D*).

Since individuals are chosen uniformly at random for strategy evaluation, a novel behavior (e.g., cooperate in an all-defector population or defect in an all-cooperator population) arises in location *i* with probability 1/N. Once the mutant appears, the population updates until the mutant type either fixes or goes extinct, and then another mutant is introduced. Thus, to quantify the evolutionary success of cooperators, we consider the probability that a mutant type takes over when placed uniformly at random within a population of residents. We denote by *ρ*_*C*_ and *ρ*_*D*_ the fixation probability of cooperation and defection, respectively. Under neutral drift (*δ* = 0), temporal interactions have no effect on these quantities since payoffs are then immaterial and both *ρ*_*C*_ and *ρ*_*D*_ are equal to 1/N. In the nontrivial case of weak selection (0<δ≪1) (13, 17, 24, 25, 29), cooperation is said to be favored relative to defection if $\rho_{C}>\rho_{D}$.

### Condition for the Evolution of Cooperation on Temporal Networks.

We first offer a theoretical condition for the evolutionary success of cooperation with temporal interactions. The evolution of cooperation with temporal interactions depends on how strategies spread among individuals, which can be studied using an ancestral process on the replacement network (Fig. 1*E*). To capture the dynamics of behavioral transmission over time, we let $P_{\left(i,j\right)}\left[T^{\text{coal}}⩽t\right]$ denote the probability that the lineages leading to *i* and *j* coalesce at most *t* ⩾ 0 time steps into the past (42), which can be calculated using single-step probabilities of random walks on the replacement network (*Materials and Methods*). The probability of moving from *i* to *j* in one step of a random walk on the replacement network is $p_{\mathrm{ij}}\;:=\;w_{\mathrm{ij}}/w_{i}$, where $w_{i}=\sum_{j=1}^{N}w_{\mathrm{ij}}$ captures the number of *i*’s neighbors, and $w_{\mathrm{ij}}=w_{\mathrm{ji}}=1$ if individuals *i* and *j* interact at least once over time (otherwise, $w_{\mathrm{ij}}=w_{\mathrm{ji}}=0$).

Cooperators are favored if they have, on average, a higher payoff than a random individual two steps away. Let $B_{n}\left(t\right)\;:=\;b\sum_{i,j,k=1}^{N}\pi_{i}p_{\mathrm{ij}}^{\left(n\right)}q_{\mathrm{jk}}\left(t\right)P_{\left(i,k\right)}\left[T^{\text{coal}}⩽t\right]$ represent the expected benefit to the individual at the end of an *n*-step random walk from a cooperator at time *t*, where $\pi_{i}\;:=\;w_{i}/\sum_{j=1}^{N}w_{j}$ defines the reproductive value (17) and $p_{\mathrm{ij}}^{\left(n\right)}$ captures the probability of moving from *i* to *j* in a *n*-step random walk. Analogously, the time-*t* derived cost to an individual at the end of an *n*-step random walk from a cooperator is $C_{n}\left(t\right)\;:=\;c\sum_{i,j=1}^{N}\pi_{i}p_{\mathrm{ij}}^{\left(n\right)}q_{j}\left(t\right)P_{\left(i,j\right)}\left[T^{\text{coal}}⩽t\right]$, where $q_{j}\left(t\right)\;:=\;\sum_{k=1}^{N}q_{\mathrm{jk}}\left(t\right)$ indicates whether individual *j* has interactions at time step *t*. Then, cooperators are favored over defectors whenever

[1] $$\begin{array}{c} -\sum_{t=0}^{\infty }C_{0}\left(t\right)+\sum_{t=0}^{\infty }B_{0}\left(t\right)>-\sum_{t=0}^{\infty }C_{2}\left(t\right)+\sum_{t=0}^{\infty }B_{2}\left(t\right), \end{array}$$

which leads to the critical benefit-to-cost ratio ${\left(b/c\right)}^{*}$ above which selection favors cooperators (*Materials and Methods*).

### The Effect of Chronological Ordering on Temporal Evolutionary Dynamics.

To understand the evolution of cooperation on naturally occurring networks, we first study temporal interactions on four empirical datasets capturing the dynamical contacts in an office building (38), a high school (39), an exhibition (43), and a hospital (44) (*SI Appendix*, Fig. S1). We construct temporal networks by aggregating social contacts over time windows of length Δt (*SI Appendix*, Figs. S2–S5). Each snapshot lasts for *N* time steps (equal to the population size), ensuring that each individual updates its strategy once, on average, over the snapshot. By randomly permuting the sequence of snapshots in each dataset, we show that the critical benefit-to-cost ratio, ${\left(b/c\right)}^{*}$, above which cooperation is favored, varies significantly, and the original ordering falls within this range (*SI Appendix*, Figs. S6–S9).

To further illustrate the effect of chronological ordering on evolutionary dynamics, we investigate temporal interactions generated using the Barabási-Albert model as a special case, where at each time step, a new node links to a certain number of existing nodes with probability proportional to their current number of neighbors (*SI Appendix*, Fig. S10*A*) (26). Due to the way that networks are constructed through preferential attachment over time, the nodes that are added first tend to have large degree, while those added later have fewer neighbors, which tends to result in the largest ${\left(b/c\right)}^{*}$, indicating the least favorable configuration for the evolution of cooperation. However, by reversing the ordering of the temporal interactions, we find a marked drop in ${\left(b/c\right)}^{*}$ relative to the natural ordering (*SI Appendix*, Fig. S10 *B* and *C*). In this reversed scheme, the individuals with fewer connections (small degree) on the network are prioritized for early interactions, and those with large degree interact later. Random orderings yield critical ratios falling in between these two extremes (*SI Appendix*, Figs. S10 and S11). The numerical results are in good agreement with our theoretical prediction given in Eq. **1** (*SI Appendix*, Fig. S10*D*).

### Evaluation of Temporal Snapshots.

**Fig. 2.**
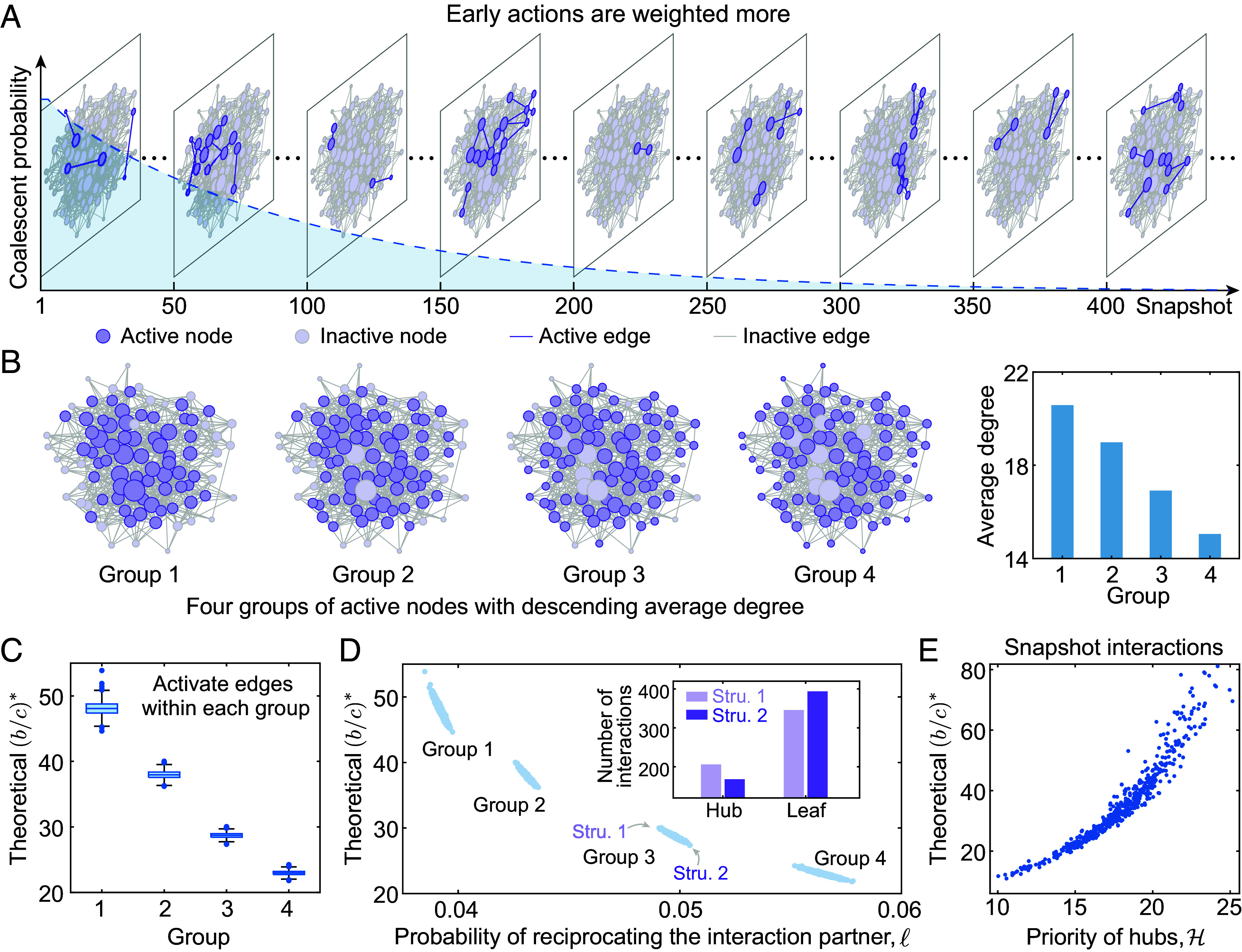
A simple rule for promoting cooperation in temporal and static networks. (*A*) We show a sequence of snapshots in empirical temporal interactions collected from the face-to-face contacts in an office building (38), where the active nodes and edges on each snapshot are marked in purple. The coalescent probability (dashed line) governing the dynamics of strategy dispersal decays exponentially, indicating that the earlier interactions are weighted more in the evolution (Eq. **3**). (*B*) We sort the nodes from largest to smallest and then generate four groups of nodes with descending node degree, with the average node degree in Group 1 to Group 4 decreasing accordingly. (*C*) The interaction edges in each group are selected with probability *P* = 0.5 from the underlying replacement networks, and the group with more hubs yields higher ${\left(b/c\right)}^{*}$. (*D*) We show ${\left(b/c\right)}^{*}$ as a function of the probability of reciprocating the interaction partner (*ℓ*). For Group 3, we divide the nodes into hubs (with top 20% degree) and leaves, and show the number of interactions accordingly on structures with the highest and lowest ${\left(b/c\right)}^{*}$ respectively, where the fewer interactions the hubs involved, the lower the value of ${\left(b/c\right)}^{*}$ is. (*E*) We calculate the critical ratio ${\left(b/c\right)}^{*}$ on each snapshot of the empirical temporal network as a function of the priority of hubs H, demonstrating that a lower priority of hubs leads to lower critical threshold for promoting the emergence of cooperation.

The impact of chronological ordering on evolutionary outcomes stems from the distinct temporal structures over time, which shape the dynamics of the strategy dispersal. So far, the problem of which interaction structure facilitates cooperation on a given base network remains poorly understood. Starting from the empirical temporal interactions collected from office contacts (38), we first investigate the evolution of cooperation with different interaction structures on each snapshot. Fig. 2*A* shows the sequence of snapshot on empirical temporal interactions over time in an office building.

To understand how different interaction structures alter the evolution of cooperation, we sort the nodes in descending order of degree on replacement networks and generate four groups of nodes subsequently (Fig. 2*B*). In this way, the former group tends to include more hubs, who have interacted with many different individuals over the whole time and have more social links compared to others. Therefore, the average node degrees in Group 1 to Group 4 decrease (Fig. 2*B*). We show that interactions among the group with more hubs generally leads to a higher value of ${\left(b/c\right)}^{*}$, which impedes the emergence of cooperation (Fig. 2*C*).

To further extract the factors determining the evolution of cooperation on different interaction structures, we simplify Eq. **1** using a mean-field approximation, obtaining the critical ratio for static interactions given by

[2] $$\begin{array}{c} {\left(\frac{b}{c}\right)}^{*}\approx \frac{\bar{\tau }q+\epsilon_{c}}{\left(1+\bar{\tau }\right)\ell -2q+\epsilon_{b}}, \end{array}$$

where $\bar{\tau }$ represents the average expected coalescence time over any pair of nodes on the replacement network, and *ϵ*_*c*_ and *ϵ*_*b*_ are negligible compared to the other quantities (*SI Appendix*, section S3). Here, $\bar{\tau }$ is fully determined for a given replacement network since that is where the ancestral process takes place. The most important factor impacting the evolution of cooperation becomes the interaction structure, which can be summarized by *q* and *ℓ*. Here, we have $q\;:=\;\sum_{i=1}^{N}\pi_{i}q_{i}$ and $\ell \;:=\;\sum_{i,j=1}^{N}\pi_{i}\left(\frac{1}{2}p_{\mathrm{ij}}q_{\mathrm{ji}}+\frac{1}{2}q_{\mathrm{ij}}p_{\mathrm{ji}}\right)$, with the time stamp *t* removed for simplification. Note that $q_{i}=1$ when individual *i* has at least one interaction, so *q* represents the sum of degrees over active nodes that have at least one interaction. By its definition, *q* is small whenever the individuals involved in interactions at time *t* have a relatively low degree in the replacement network. This property indicates that less participation of hubs can result in a lower critical ratio, which is confirmed by our theoretical calculations of ${\left(b/c\right)}^{*}$ over Group 1 to Group 4 in Fig. 2*C*.

By activating edges randomly from the underlying network, we show an independent effect of the other factor, *ℓ*, on the evolution of cooperation, which captures the probability of reciprocating the strategy of the interaction partner (Fig. 2*D*). Intuitively, it indicates the mean probability of moving first from *i* to a neighbor and then back to *i* in the subsequent step. In this way, interactions between low-degree nodes also lead to higher probabilities of imitating the strategy from interaction partners. For given nodes in the static interaction networks, ${\left(b/c\right)}^{*}$ decreases when the probability of reciprocating with the interaction partner (*ℓ*) increases within each group of nodes (Fig. 2*D*). We further show that the number of interactions of hubs on the favorable interaction structure in Group 3 decreases compared to that with the largest ${\left(b/c\right)}^{*}$. This also confirms that individuals with fewer social connections should be more involved in interactions.

Taken together, these two dominant factors show how the interaction structure may promote the evolution of cooperation when hubs are less involved. Specifically, we offer a general index, H := q/ℓ, which represents the priority of the hubs and is based on the degree to which hubs are involved in interactions. By calculating the critical ratio on each snapshot of the empirical interactions, we illustrate a positive association between H and ${\left(b/c\right)}^{*}$ in Fig. 2*E*. This indicates that the degree to which the interaction structure favors the emergence of cooperation can be evaluated by hub priority, where the lower H leads to the lower critical threshold for favoring cooperation.

Another intuitive example is based on temporal interactions generated using the Barabási-Albert model (26) (*SI Appendix*, Fig. S12*A*). Since the natural ordering of temporal networks generated by preferential attachment involves more hubs earlier on, we can obtain the groups with descending average nodes degree by dividing the sequence of subsequent snapshots evenly (*SI Appendix*, Fig. S12*B*). Accordingly, we can obtain consistent results by evaluating the interactions within each group with the extracted dominant factors *q* and *ℓ* (*SI Appendix*, Fig. S12 *C* and *D*).

### Promoting Cooperation with Temporal Interactions.

With this understanding of snapshot interaction structures, we are now able to explore how time sequences regulate different snapshots from a macroscopic perspective. The key point of how time acts on behavioral transmission in the evolution of cooperation lies in the coalescent probability in the ancestral process. Let *τ* denote the meeting time for independent random walks. Here, we use $P\left[\tau =m\right]$ to capture the expected probability that two random walks starting from any pair of different nodes, separately, and meet after exactly *m* time steps, where in each time step, exactly one walk is selected (uniformly at random) to take a step (*Materials and Methods*).

We uncover that the correspondence between every single snapshot structure and the overall sequence of temporal interactions, as well as how they combine to play a role in the evolution, are captured by the coalescent probability, $P\left[\tau =m\right]$ (Fig. 2*A*). With a similar form in Eq. **2** and simplified from Eq. **1**, the critical ratio for favoring cooperation with any temporal interactions is

[3] $$\begin{array}{c} {\left(\frac{b}{c}\right)}^{*}\approx \frac{Q+\varepsilon_{c}}{L-2\bar{q}+\varepsilon_{b}}. \end{array}$$

Here, $Q\;:=\;\sum_{m=1}^{\infty }\sum_{T=0}^{m-1}q\left(\left\lfloor TN/2\right\rfloor \right)P\left[\tau =m\right]$ defines the accumulated time-averaged reproductive value of active nodes (*q*) over the snapshot sequence, and $\bar{q}\;:=\;\sum_{m=0}^{\infty }q\left(\left\lfloor mN/2\right\rfloor \right)P\left[\tau =m\right]$ represents the time-averaged *q*, which is positively related to *Q*. Analogously, $L\;:=\;\sum_{m=1}^{\infty }\sum_{T=0}^{m}\ell \left(\left\lfloor TN/2\right\rfloor \right)P\left[\tau =m\right]$ indicates the accumulated time-averaged probability of reciprocating with the interaction partner (*ℓ*). This theoretical prediction holds with remarkable accuracy on both empirical and synthetic temporal networks (*SI Appendix*, Figs. S13 and S14).

We show that early interaction structures are weighted more in the critical ratio than later snapshots in Eq. **3**, since $P\left[\tau =m\right]$ decays exponentially in *m* (Fig. 2*A* and *SI Appendix*, Fig. S15). Intuitively, the behavioral transmission converges rapidly, and therefore earlier interactions have more effect on the process of strategy dispersal. In particular, lower values of ${\left(b/c\right)}^{*}$ are achieved by structures for which *L* is large relative to *Q*, so interaction structures with small average reproductive value ($q\left(t\right)$) and large probability of reciprocating the strategy from interaction partners ($\ell \left(t\right)$) should occur early. Combined with our findings on the snapshot structure, the low-degree nodes should be involved in interactions earlier, while hubs should be temporally deprioritized, namely a low hub priority, H, should be realized earlier to facilitate the emergence of cooperation.

A natural example for interpreting the role of hubs in evolutionary dynamics is the temporal networks generated by preferential attachment. The way in which these temporal networks are constructed determines the two extreme ${\left(b/c\right)}^{*}$ in cases of natural and reversed orderings in the evolution of cooperation, with random permuted sequences falling in between (*SI Appendix*, Figs. S10 and S11). With hubs substantially involved in earlier interactions, the natural ordering presents the largest critical ratio, ${\left(b/c\right)}^{*}$ (*SI Appendix*, Figs. S10 and S11), while the reversed ordering results in the lowest value of ${\left(b/c\right)}^{*}$ due to the sufficient interactions of small nodes in earlier interactions (*SI Appendix*, Figs. S10 and S11). We further show that this result is robust for large networks with $N=10^{4}$ (*SI Appendix*, Fig. S16), and it can be extended to a fixed-cost, fixed-benefit model representing divisible social goods (*SI Appendix*, Fig. S17).

Moreover, we show that our conclusion can also be applied to other update rules, including imitation and pairwise-comparison mechanisms (*SI Appendix*, section S4). For imitation updating, the theoretical critical ratio shares the same form of that of death-birth updating. Therefore, a design approach of using the hub priority, H, to rearrange the ordering of sequences can be extended to the imitation rule (*SI Appendix*, Fig. S18). Another natural way to confirm our conclusion in other processes is to apply the temporal networks generated by preferential attachment, which have correlations between degree sequence and the order of appearance. For both imitation and pairwise-comparison updating, we show consistent results illustrating the reversed ordering (in which hubs are temporally deprioritized) can promote cooperation (*SI Appendix*, Fig. S19).

### Designing Optimal Temporal Interactions on Empirical Datasets.

**Fig. 3.**
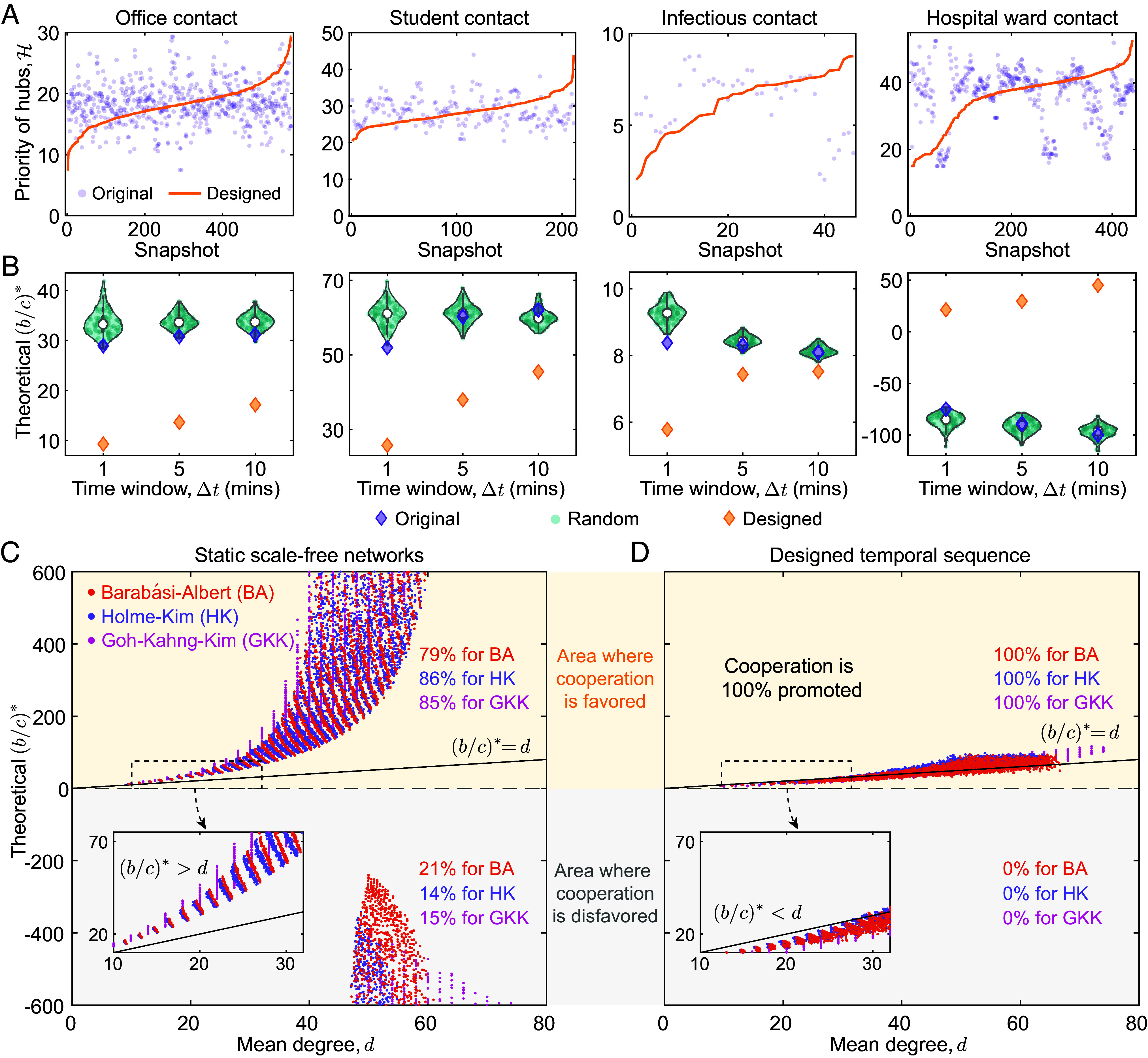
Designing temporal interactions on empirical and synthetic networks. (*A*) We show the priority of hubs over snapshots with the original (purple dot) and designed (golden line) ordering in four empirical datasets collected from contacts in office (38), high school (39), exhibition (43), and hospital (44), respectively. The corresponding temporal networks are constructed by aggregating interactions with time window of Δt=10 min. The original empirical networks exhibit temporal randomness in the priority of hubs over the snapshots, which are rearranged in ascending ordering in the designed sequences. (*B*) Correspondingly, we calculate the theoretical value of critical threshold ${\left(b/c\right)}^{*}$ on temporal interactions with both natural (purple diamond) and designed (golden diamond) ordering on those four empirical datasets. As a control group, we plot the distribution of the random permuted snapshot sequence over 100 samples in cyan dots and shadows. For different values, Δt, our designed ordering of interactions facilitates the emergence of cooperation compared to both original and randomized interaction sequences. (*C*) We show scatter plot of the critical threshold, ${\left(b/c\right)}^{*}$, based on mean degree, *d*, on Barabási-Albert (BA) (26), Holme-Kim (HK) (45), and Goh-Kahng-Kim (GKK) (46) scale-free networks. Each type of network has negative ${\left(b/c\right)}^{*}$, which implies that spite can be favored and there is no possibility for those networks to favor cooperation. (*D*) With our designed temporal interactions, all scale-free networks have positive ${\left(b/c\right)}^{*}$, which is approximately equal to the average degree (*d*) of the network. All structures have $10^{4}$ realizations, with the number of nodes 100⩽N⩽150 and mean degree 10⩽d⩽100 for each network.

Using the intuition gathered so far, we now shift our focus back to empirical data, and ask whether our key predictions can be validated on empirical temporal interactions. We calculate the hub priority, H, over snapshots on empirical temporal interactions, and show that the priority of hubs in the original sequences are disordered and stochastic over time (purple dots in Fig. 3*A*) in all empirical datasets. Our design approach is to rearrange the snapshots of the sequence in an ascending ordering of H (golden lines in Fig. 3*A* and *SI Appendix*, Figs. S2–S5). As a comparison, Fig. 3*B* shows that ${\left(b/c\right)}^{*}$ for the original ordering falls within the range of those in the random order by randomly permuting the sequence of snapshots. This further confirms that the extent to which hubs are involved in the interactions tends to be temporally disordered in real networks, which also opens up the possibility of designing the ordering of interaction sequences.

By rearranging the sequence in the ascending ordering of the hub priority, we find that the emergence of cooperation is greatly facilitated for all empirical datasets (Fig. 3*B*). In cases for which ${\left(b/c\right)}^{*}>0$ for contacts in office (38), high school (39) and exhibition (43) (meaning cooperation can evolve at all), we show the reordered sequence results in a lower value of ${\left(b/c\right)}^{*}$ for different values of Δt (Fig. 3*B* and *SI Appendix*, Figs. S6–S8). Remarkably, for the original network collected from contacts in hospital (44) with ${\left(b/c\right)}^{*}<0$ [which means that spite is favored (17)], the reordered sequence results in a positive value of ${\left(b/c\right)}^{*}$, presenting a new avenue for cooperation to evolve (Fig. 3*B* and *SI Appendix*, Fig. S9). In all scenarios, the designed snapshot ordering enhances the emergence of cooperation, relative to the original and random permuted interaction sequences on empirical networks.

In many practical situations, the exact timing of the interactions is unknown. By taking each edge on the static network as an interaction snapshot, the temporal network can be arranged in ascending ordering of hub priority, H. Fig. 3 *C* and *D* present the critical threshold ${\left(b/c\right)}^{*}$ on designed temporal interactions based on three different types of scale-free networks. We find that although sparse scale-free networks require a high critical ratio, with ${\left(b/c\right)}^{*}$ larger than the mean degree *d* (Fig. 3*C*), orchestrated temporal interactions on scale-free networks are more favorable for cooperation, with ${\left(b/c\right)}^{*}<d$ (Fig. 3*D*). Furthermore, 100% of scale-free networks with orchestrated temporal interactions have positive values of ${\left(b/c\right)}^{*}$ and present significant advantages for altruistic behaviors (Fig. 3*D* and *SI Appendix*, Fig. S20). Our results demonstrate the striking role of temporal interactions on highly heterogeneous networks, especially in scenarios where the corresponding static (aggregated) networks are dense.

### Temporal Interactions with Bursty Patterns.

**Fig. 4.**
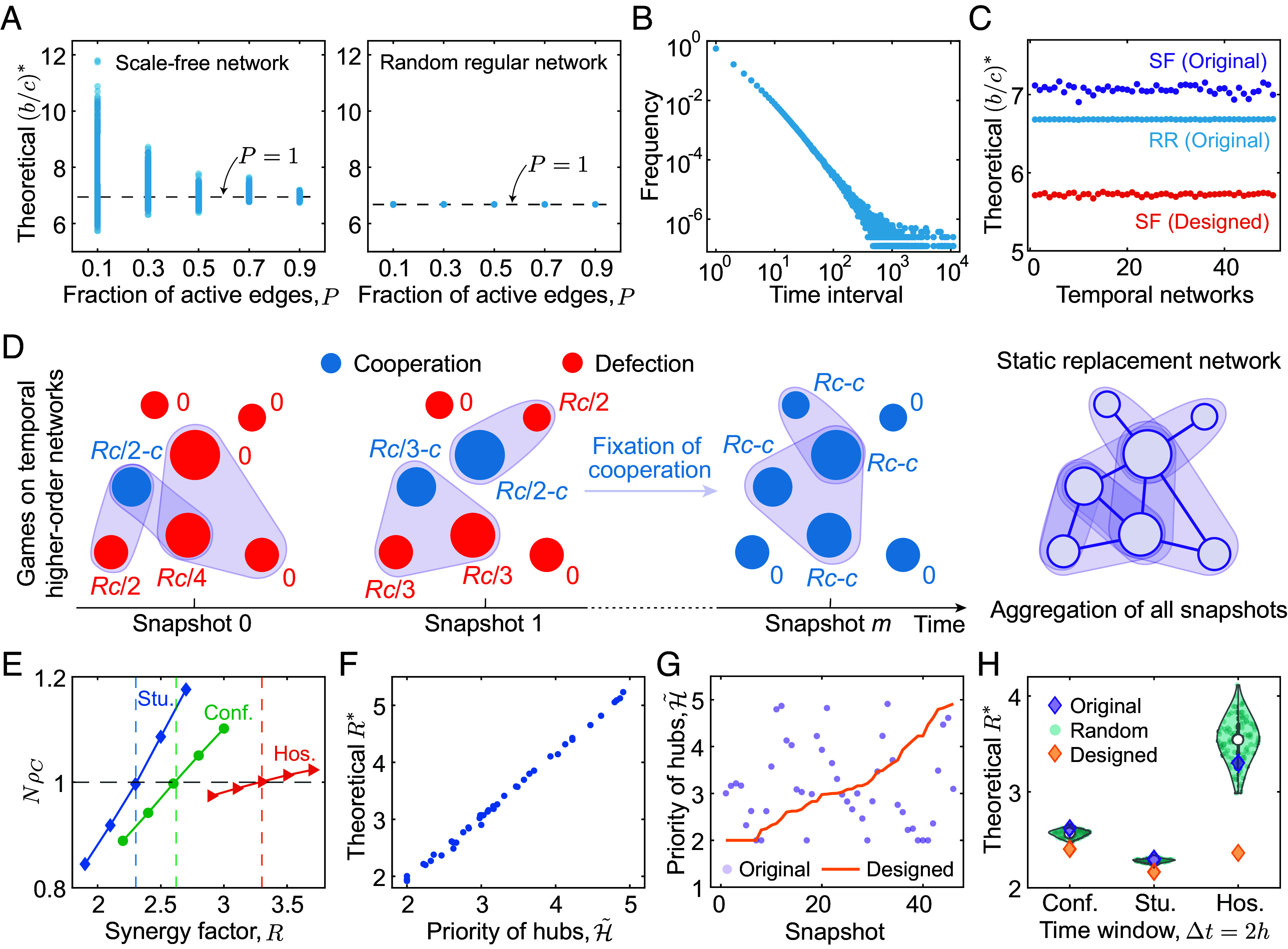
Designing temporal interactions with bursty patterns and higher-order networks. (*A*) We present the critical benefit-to-cost ratio ${\left(b/c\right)}^{*}$ on interaction networks generated by randomly choosing a fraction, *P*, of edges from scale-free (*Left* panel) and random regular (*Right* panel) networks with *N* = 100. Each dot corresponds to an interaction network, and 100 samples are plotted over each value of *P*. (*B*) We generate temporal interactions with bursty patterns on random regular networks (47), and present the power-law distribution of the interevent time intervals on links. (*C*) We calculate ${\left(b/c\right)}^{*}$ on 50 independent sequences of temporal interactions with $10^{5}$ time steps on both scale-free and random regular networks, and further design the sequence on scale-free networks to reduce the critical ratio. (*D*) We illustrate evolutionary games on temporal higher-order networks, where individuals play public goods games on hyperedges (light purple) at each time step. The aggregated static replacement networks capture the social relationships (purple links), where each individual has connections to those who have interacted within a common group at least once over time. (*E*) We numerically show the fixation probability of cooperation (*ρ*_*C*_) as a function of the synergy factor ratio (*R*) on temporal higher-order interactions constructed from empirical contacts in high school (Stu.) (39), conference (Conf.) (43), and hospital (Hos.) (44). The critical synergy factor, $R^{*}$, is further predicted by the theoretical calculations (vertical dashed line) using *SI Appendix*, Eq. **54**. (*F*) We define the priority of hubs $\tilde{H}$ on higher-order interactions, and show its relationship to the theoretical value of $R^{*}$ on snapshots of hospital contacts. We further design the sequence of interactions by rearranging the original sequence in ascending order of $\tilde{H}$ [golden line in (*G*)], and calculate the $R^{*}$ on temporal higher-order interactions with original (purple diamond), random (100 samples in cyan dots), and designed (golden diamond) ordering on those three empirical datasets in (*H*).

In addition to empirical temporal interactions and synthetic networks generated by preferential attachment, one may ask whether our conclusion holds on interactions with bursty patterns, which is commonly studied in human dynamics. To answer this question, we start from investigating interactions on typical heterogeneous and homogeneous networks: scale-free and random regular. By generating interaction networks with randomly activating edges at fraction *P*, we show that the effects of different interaction structures on heterogeneous networks are far more complex than those on homogeneous networks. Different interaction structures strongly influence ${\left(b/c\right)}^{*}$ on scale-free networks, especially at small values of *P*, but this value is nearly unaffected by *P* on random regular networks (Fig. 4*A*). However, the random activation of a fraction of edges at each time step naturally leads to a succession of interactions with relatively homogeneous interevent times.

To explore the evolutionary dynamics on interaction structures with bursty patterns, we construct temporal interactions with heavy-tailed distributions of the link activity patterns on both homogeneous and heterogeneous networks (47). Fig. 4*B* shows that the frequency of interevent time intervals on links obeys a power-law distribution, based on which interactions occur with a diverging time scale and exhibit bursty patterns. We show that random regular networks are still unaffected even with bursty interactions (Fig. 4*C*). Moreover, we prove that for any interactions on homogeneous structures, the critical ratio ${\left(b/c\right)}^{*}$ approaches the average degree on the underlying networks (*SI Appendix*, section S3). In contrast, heterogeneous networks show variations of ${\left(b/c\right)}^{*}$ over different realizations of temporal bursty interactions. We further confirm the effectiveness of our proposed design approach by rearranging the orderings of snapshots with ascending order of the hub priority, H, where the designed interaction sequence on scale-free networks present remarkable drops in ${\left(b/c\right)}^{*}$ compared to that of the original counterparts (Fig. 4*C*).

### Temporal Higher-Order Networks.

With the rise of network science, a number of studies have emerged in recent years focusing on higher-order network structures (48–50), which are known to capture group interactions among individuals. Here, we extend our model to temporal hypernetworks, where higher-order interactions within groups at each snapshot are captured by hyperedges (Fig. 4*D*). At each time step, individuals participate in public goods games on all hyperedges, where cooperators pay a cost for each game and defectors pay nothing (27). Individuals then receive the returns from the common pool, where the total investment from all cooperators is enhanced by a synergy factor *R* and then divided evenly among the groups. After each round of games, a random individual is chosen to update and imitate the strategy from its neighbors on the strategy replacement networks, with probability proportional to the corresponding fitness. Note that the underlying replacement networks are obtained by aggregating all hypernetworks over time and connecting each pair of nodes within each hyperedge. Therefore, a hub with many social links indicates that it has group interactions with many different individuals over time (Fig. 4*D*).

We calculate the theoretical critical synergy factor, $R^{*}$ (dashed lines), above which cooperation is favored, on three empirical temporal higher-order interactions collected from high school (Stu.) (39), conference (Conf.) (43), and hospital (Hos.) (44) datasets, and confirm the accuracy of our predictions with numerical simulations of fixation probabilities, *ρ*_*C*_ (solid lines) (Fig. 4*E*). The formal proof of the theoretical results is given in *SI Appendix*, section S5. Similar to pairwise interactions, we can define a priority measure of the hubs, $\tilde{H}\;:=\;q/\sum_{i=1}^{N}\pi_{i}{\tilde{q}}_{\mathrm{ii}}$, for the evaluation of the interaction structures. Here, ${\tilde{q}}_{\mathrm{ii}}$ indicates the probability of subsequent random walks from nodes *i*, moving to all hyperedges containing *i*, and back to itself. Taken together, we show that the hub priority, $\tilde{H}$, takes lower values with fewer hubs involved (smaller *q*), and smaller interaction groups (larger ${\tilde{q}}_{\mathrm{ii}}$). Analogously, we illustrate a positive association between the critical synergy factor, $R^{*}$, and the hub priority, $\tilde{H}$, for hypernetworks (Fig. 4*F*). Moreover, this measure can be further used to design the orderings of temporal hypernetworks by rearranging the snapshots in ascending order of $\tilde{H}$ (Fig. 4*G*). By designing temporal orderings for empirical hypernetworks, we demonstrate a reduction in $R^{*}$ compared to original interactions over different empirical datasets (Fig. 4*H*). In general, our proposed design framework remains effective on empirical temporal higher-order interactions.

## Discussion

There is a large body of important research exploring the evolution of cooperation on dynamic networks, including coevolutionary games, where network structures coevolve with individual strategies (51–53). In these endogenous dynamic networks, structural changes are determined by individual decisions to improve the environment pertaining to interactions and games. In contrast, here we focus on exogenous temporal interactions collected from empirical contacts, where changes of network structures are independent of the evolutionary games. Our framework extends to cover strategy dynamics on publicly available empirical datasets recording real temporal interactions (Fig. 3).

The simple rule we reported here provides a unifying understanding of the evolution of cooperation with temporal interactions and how it relates to evolutionary dynamics on static networks. Previous studies have reported that cooperation can be promoted in low-degree networks because it indicates high social relatedness (13), and hubs with many social ties tend to decrease social relatedness. By temporally deprioritizing the interactions of hubs, we may effectively increase the relatedness earlier during the course of social evolution, which lowers the threshold for collective cooperation to emerge on heterogeneous networks. In this way, our findings circumvent the limitations of previous findings on static networks, which have shown that heterogeneous networks require a higher threshold for boosting cooperation than homogeneous networks (13, 17, 54). By designing temporal interactions for the underlying heterogeneous networks, we may take the fundamental advantages of temporal networks to promote collective cooperation (Fig. 3*D* and *SI Appendix*, Figs. S20 and S21).

Another promising application points to the design of interaction sequences in practical scenarios. The empirical temporal networks studied here illustrate interpersonal interactions at each point in time (38, 39, 43, 44). Our work provides a concise and efficient metric to arrange interaction sequences—sorting the sequence in the ascending ordering of hub priority for each snapshot. For instance, meeting times between different teams in an office can be rearranged to maximize cooperative behaviors among team members. Hospitals can arrange and prioritize some designated patients for consultation in their scheduling. By arranging the sequence of interactions according to the guidelines provided here, cooperation can be significantly promoted (Fig. 3*B* and *SI Appendix*, Figs. S6–S9). Our findings thus extend insights into the evolution of cooperation to temporal networks, which is and will continue to be a focal topic in modern science.

## Materials and Methods

### Evolutionary Process.

Individuals obtain their payoffs via playing games with their interaction partners at each time step. Individual *i* has $I_{i}\left(t\right)\;:=\;\sum_{j=1}^{N}I_{\mathrm{ij}}\left(t\right)$ interaction partners at time *t*, where $I_{\mathrm{ij}}\left(t\right)=I_{\mathrm{ji}}\left(t\right)=1$ indicates that there is an interaction between players *i* and *j* at the current time step (and $I_{\mathrm{ij}}\left(t\right)=I_{\mathrm{ji}}\left(t\right)=0$ otherwise). The payoff matrix of the game is given by 
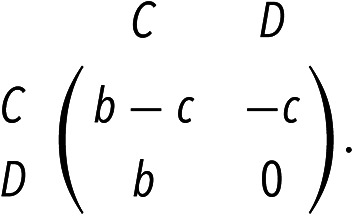


The state of network at any given time can be encoded by a binary vector $x\in {\left\{0,1\right\}}^{N}$, where $x_{i}=1$ denotes that the individual *i* chooses strategy *C*; otherwise, $x_{i}=0$ indicates *D*. Using this representation of the network state **x**, the average payoff for individual *i* is $f_{i}\left(x,t\right)=-cq_{i}\left(t\right)x_{i}+b\sum_{k=1}^{N}q_{\mathrm{ik}}\left(t\right)x_{k}$, where $q_{\mathrm{ik}}\;:=\;I_{\mathrm{ik}}\left(t\right)/I_{i}\left(t\right)$ indicates the probability of moving from *i* to *k* in one step of a random walk on the snapshot at time *t* when $I_{i}\left(t\right)>0$ (and $q_{\mathrm{ik}}\left(t\right)\;:=\;0$ otherwise). After each round of the game, an individual *i* is randomly chosen to copy the strategy from *j*, who is the neighbor of *i* on the replacement network. The probability for a node *j* to transmit its strategy to *i* at time *t* is

[4] $$\begin{array}{c} e_{\mathrm{ji}}\left(x,t\right)=\frac{1}{N}\frac{w_{\mathrm{ij}}F_{j}\left(x,t\right)}{\sum_{k=1}^{N}w_{\mathrm{ik}}F_{k}\left(x,t\right)}, \end{array}$$

where $F_{j}\left(x,t\right)$ indicates the fitness of individual *j*.

### The Condition of Evolutionary Success.

We now consider the intuitive idea of calculating the expected benefit $B_{n}\left(t\right)$ in Eq. **1** based on the ancestral process by taking the initial cooperator at location *i* as the ancestor. Starting from the stationary distribution, $\pi_{i}\;:=\;w_{i}/\sum_{j=1}^{N}w_{j}$, which defines the reproductive value (17), we transition to *j* in an *n*-step random walk with probability $p_{\mathrm{ij}}^{\left(n\right)}$. The way that individual *j*—who is *n*-steps away from the cooperative ancestor *i*—receives a benefit from its neighbor *k* relies on the condition that *k* cooperates with *j* at time *t*. This implies that first there should be an interaction between *j* and *k* at time *t*, so that *k* can contribute a benefit to the average payoff obtained by *j*, which is captured by the one step probability $q_{\mathrm{jk}}\left(t\right)$ of moving from *j* to *k*. Then individual *k* should have already traced its ancestry to the same lineage as the cooperator *i* with probability $P_{\left(i,k\right)}\left[T^{\text{coal}}⩽t\right]$ (so that they share the same strategy spread from the common ancestor), and *k* is a cooperator at time *t*. The calculation of the expected cost $C_{n}\left(t\right)$ in Eq. **1** is analogous to that of $B_{n}\left(t\right)$. The primary difference between $B_{n}\left(t\right)$ and $C_{n}\left(t\right)$, apart from the factors *b* and *c*, is that $C_{n}\left(t\right)$ depends on $P_{\left(i,j\right)}\left[T^{\text{coal}}⩽t\right]$ instead of $P_{\left(i,k\right)}\left[T^{\text{coal}}⩽t\right]$, owing to the fact that, whenever *j* is a cooperator, *j* incurs a cost for each interaction partner *k* at time *t*.

At a higher level, this echoes the same intuition for the corresponding condition on static networks (17), with the reasoning being that cooperators compete with two-step neighbors to have their strategy imitated by a common neighbor. Since summing $B_{n}\left(t\right)$ and $C_{n}\left(t\right)$ over all *t* in Eq. **1** generally leads to divergent series, we replace $P_{\left(i,j\right)}\left[T^{\text{coal}}⩽t\right]$ by $1-P_{\left(i,j\right)}\left[T^{\text{coal}}>t\right]$ and cancel out the common factor in Eq. **1** to mitigate this issue. Then we obtain

[5] $$\begin{array}{cc} \sum_{t=0}^{\infty }B_{0}\left(t\right)-\sum_{t=0}^{\infty }B_{2}\left(t\right) & =b\left(\sum_{t=0}^{\infty }\sum_{i,j,k=1}^{N}\pi_{i}p_{\mathrm{ij}}^{\left(2\right)}q_{\mathrm{jk}}\left(t\right)P_{\left(i,k\right)}\left[T^{\text{coal}}>t\right]\right.\\ & -\left.\sum_{t=0}^{\infty }\sum_{i,j=1}^{N}\pi_{i}q_{\mathrm{ij}}\left(t\right)P_{\left(i,j\right)}\left[T^{\text{coal}}>t\right]\right) \end{array}$$

and

[6] $$\begin{array}{cc} & \sum_{t=0}^{\infty }C_{0}\left(t\right)-\sum_{t=0}^{\infty }C_{2}\left(t\right)\\ & =c\sum_{t=0}^{\infty }\sum_{i,j=1}^{N}\pi_{i}p_{\mathrm{ij}}^{\left(2\right)}q_{j}\left(t\right)P_{\left(i,j\right)}\left[T^{\text{coal}}>t\right]. \end{array}$$

The critical benefit-to-cost ratio ${\left(b/c\right)}^{*}$ above which cooperation is favored can be calculated by applying Eq. **1**. In *SI Appendix*, sections S1–S3, we give a formal proof of this result and demonstrate how to evaluate it.

### Coalescent Probability.

We consider the ancestral process going backward in time, defined by the neutral replacement rule in Eq. **4**, and the population starts from a single uniformly selected cooperator. Any subset of nodes will eventually descend from a common ancestor, and we let $T^{\text{coal}}$ be the number of steps backward until this happens for the first time. Given starting nodes of *i* and *j*, we denote by $P_{\left(i,j\right)}\left[T^{\text{coal}}=t\right]$ the probability that it takes exactly *t* steps for *i* and *j* to descend from a common ancestor in the resulting coalescing random walk. Based on Eq. **4**, these terms can be calculated using the recurrence

[7] $$\begin{array}{c} P_{\left(i,j\right)}\left[T^{\text{coal}}=t\right]=\left\{\begin{array}{cc} \frac{1}{N}\sum_{k=1}^{N}p_{\mathrm{ik}}P_{\left(k,j\right)}\left[T^{\text{coal}}=t-1\right]\\ +\frac{1}{N}\sum_{k=1}^{N}p_{\mathrm{jk}}P_{\left(i,k\right)}\left[T^{\text{coal}}=t-1\right]\\ +\frac{N-2}{N}P_{\left(i,j\right)}\left[T^{\text{coal}}=t-1\right], & i\neq j\\ 0, & i=j \end{array}\right. \end{array}$$

when *t* > 0. When *t* = 0, we have $P_{\left(i,j\right)}\left[T^{\text{coal}}=0\right]=1$ if *i* = *j* and 0 otherwise. By summing both side of the above equation from $P_{\left(i,j\right)}\left[T^{\text{coal}}=t+1\right]$ to infinity, we obtain the probability that two random walks starting from *i* and *j* have not coalesced by time step *t*, which is given by

[8] $$\begin{array}{c} P_{\left(i,j\right)}\left[T^{\text{coal}}>t\right]=\left\{\begin{array}{cc} \frac{1}{N}\sum_{k=1}^{N}p_{\mathrm{ik}}P_{\left(k,j\right)}\left[T^{\text{coal}}>t-1\right]\\ +\frac{1}{N}\sum_{k=1}^{N}p_{\mathrm{jk}}P_{\left(i,k\right)}\left[T^{\text{coal}}>t-1\right]\\ +\frac{N-2}{N}P_{\left(i,j\right)}\left[T^{\text{coal}}>t-1\right], & i\neq j\\ 0, & i=j \end{array}\right. \end{array}$$

when *t* > 0. And $P_{\left(i,j\right)}\left[T^{\text{coal}}>0\right]=0$ if *i* = *j* and 1 otherwise. By calculating $P_{\left(i,j\right)}\left[T^{\text{coal}}>t\right]$ with the above equation, we obtain the critical benefit-to-cost ratio in Eq. **1**.

At each time step during the evolutionary process, one of the two random walkers is chosen to take a step of random walk with probability 2/N. We now only consider two independent random walks starting from *i* and *j* on the replacement network with meeting time *τ*, where each of the random walkers is chosen with equal probability (1/2) at each time step (Fig. 1*E*). The probability that *i* and *j* coalesce in exact *m* steps follows recurrence relation of

[9] $$\begin{array}{c} P_{\left(i,j\right)}\left[\tau =m\right]=\left\{\begin{array}{cc} \frac{1}{2}\sum_{k=1}^{N}p_{\mathrm{ik}}P_{\left(k,j\right)}\left[\tau =m-1\right]\\ +\frac{1}{2}\sum_{k=1}^{N}p_{\mathrm{jk}}P_{\left(i,k\right)}\left[\tau =m-1\right] & i\neq j\\ 0 & i=j \end{array}\right. \end{array}$$

for *m* > 0. And $P_{\left(i,j\right)}\left[\tau =0\right]=1$ if *i* = *j* and 0 otherwise. By rewriting Eq. **1** with $P_{\left(i,j\right)}\left[\tau >m\right]$ and applying the above recurrence relation, we replace $P_{\left(i,j\right)}\left[\tau =m\right]$ by the mean-field approximation $P\left[\tau =m\right]$ when *i* ≠ *j*, and obtain the results in Eq. **3** (*SI Appendix*, section S3).

## Supplementary Material

Appendix 01 (PDF)

## Data Availability

All study data are included in the article and/or *SI Appendix*.
