# Supplementary material for: Promoting collective cooperation through temporal interactions
Source: Proc Natl Acad Sci U S A. 2025 Jun 27;122(26):e2509575122. doi: 10.1073/pnas.2509575122 (PMC12232700; doi:10.1073/pnas.2509575122)
Supplement: Supplementary file 1 — Appendix 01 (PDF) [file pnas.2509575122.sapp.pdf]

PNAS

Supporting Information for

Promoting collective cooperation through temporal interactions

Yao Meng, Alex McAvoy and Aming Li

Aming Li.
E-mail: amingli@pku.edu.cn

This PDF file includes:

Supporting text
Figs. S1 to S21
SI References

Supporting Information Text

S1. Model and notation

Graph. The population has exactly N individuals, and interactions among these individuals are represented by undirected, weighted, temporal networks, where nodes indicate individuals and edges represent who interacts with whom in a given time step. At time $t \in \{0, 1, 2, \ldots\}$, the intensity of the interaction between i and j is represented by a weight $I_{ij}(t) \geqslant 0$. The weighted interaction degree for node i at time t is the summation $I_i(t) \coloneqq \sum_{j=1}^{N} I_{ij}(t)$, where $I_i(t) = 0$ indicates that individual i has no interactions at time t.

The replacement of strategies occurs on an undirected, weighted, *static* replacement network. In the main text, we construct such a replacement network by aggregating snapshots of the interaction network, but, here, for the sake of generality in our mathematical results, we allow the replacement network to be arbitrary. Let $\left(w_{ij}\right)_{i,j=1}^{N}$ be such a network, which we assume is connected and satisfies $w_{ij} = w_{ji}$ for all i and j. The total weighted degree of node i is $w_i \coloneqq \sum_{j=1}^{N} w_{ij}$, and we denote the sum of all such degrees by $W \coloneqq \sum_{i=1}^{N} w_i$.

The probability of moving from i to j in one step of a random walk on the replacement network is $p_{ij} \coloneqq w_{ij}/w_i$. More generally, we denote by $p_{ij}^{(n)}$ the probability of moving from i to j in n steps of a random walk on this network. In the large-n limit, we have $\lim_{n\to\infty} p_{ij}^{(n)} = w_j/W$ (1).

Analogously, in a random walk on the interaction network at time t, the probability of moving from i to j is

$$q_{ij}(t) \coloneqq \begin{cases} \dfrac{I_{ij}(t)}{I_i(t)} & I_i(t) > 0, \\ 0 & I_i(t) = 0. \end{cases} \qquad [1]$$

Payoff. At each point in time, the individuals in the population each have one of two actions: cooperate (C) or defect (D). The state of the evolutionary population is thus given by $\mathbf{x} \in \{0, 1\}^N$, where $x_i = 1$ indicates that individual i is a cooperator and $x_i = 0$ indicates that i is a defector. When two such individuals interact via a shared link, they receive payoffs based on the matrix

$$\begin{array}{c} \\ C \\ D \end{array} \begin{array}{c} \begin{array}{cc} C & D \end{array} \\ \begin{pmatrix} b-c & -c \\ b & 0 \end{pmatrix} \end{array}. \qquad [2]$$

This "donation game" describes an interaction in which a cooperator pays c to provide a benefit b to the co-player, and defectors do nothing (2). When $b > c > 0$, as we assume here, this game is an instance of a prisoner's dilemma. At time t, all individuals interact with their neighbors on the current snapshot of the interaction graph. The payoffs for these interactions are then combined into a net payoff, which, at time t and state $\mathbf{x}$, is

$$u_i(\mathbf{x}, t) = -c q_i(t) x_i + b \sum_{j=1}^{N} q_{ij}(t) x_j, \qquad [3]$$

where $q_i(t) = \sum_{j=1}^{N} q_{ij}(t)$. The "fitness" of individual i is

$$F_i(\mathbf{x}, t) \coloneqq e^{\delta u_i(\mathbf{x},t)} = 1 + \delta u_i(\mathbf{x}, t) + O\left(\delta^2\right), \qquad [4]$$

where $\delta \geqslant 0$ is a parameter representing the intensity of selection (3). Neutral drift is modeled by $\delta = 0$, in which case the payoffs are irrelevant, and weak selection is captured by $0 < \delta \ll 1$.

Evolutionary dynamics. The evolutionary process is modeled as a discrete-time (inhomogeneous) Markov chain on $\{0, 1\}^N$, where the state is updated through replacement events. Here, we extend formal notions of replacement events and update rules (4, 5) to cover time-inhomogeneous updates, which is necessary due to the temporally varying nature of the contact networks. A replacement event is simply a pair (R, α), where $R \subseteq \{1, \ldots, N\}$ is the subset of individuals who are replaced and $\alpha : R \to \{1, \ldots, N\}$ is the offspring to parent map, such that individual $i \in R$ is replaced by the offspring of $\alpha(i)$. Time-inhomogeneity enters into the picture through how the pair (R, α) is chosen. At time t, in state $\mathbf{x} \in \{0, 1\}^N$, the pair (R, α) is chosen with probability $p_{(R,\alpha)}(\mathbf{x}, t)$.

Specific details on how temporal networks determine these replacement probabilities are illustrated below for death-Birth updating. However, for the time being, we are deliberately agnostic about how specifics of the process enter into the expressions, as we aim to derive some of our results in as broad a degree of generality as possible before specializing the model. To this end, we make the assumption that when $\delta = 0$, indicating neutral drift, $p_{(R,\alpha)}(\mathbf{x}, t)$ is independent of both $\mathbf{x}$ and t, in which case we denote this quantity by $p^{\circ}_{(R,\alpha)}$. We use the superscript $\circ$ to denote that a quantity is associated to neutral drift. The only other derived quantity we will make extensive use of is the marginal probability that i transmits its offspring to j, defined by

$$e_{ij}(\mathbf{x}, t) \coloneqq \sum_{\substack{(R,\alpha) \\ j \in R,\ \alpha(j) = i}} p_{(R,\alpha)}(\mathbf{x}, t). \qquad [5]$$

By our assumption, $e_{ij}(\mathbf{x}, t)$ is independent of $\mathbf{x}$ and t when $\delta = 0$, and we denote this quantity by e°_{ij}.

S2. Fixation probabilities in time-inhomogeneous models

From studies of weak selection, three important considerations have emerged: reproductive value, assortment under neutral drift, and coefficients showing how the game (selection) affects replacement dynamics (5). Here, we focus on how each of these aspects is incorporated into the model.

Since the replacement network is static and the interactions do not affect dynamics under neutral drift, reproductive value (a neutral quantity) plays the same role here as it has in previous studies not involving time-inhomogeneity (4–9). The reproductive value (RV) of i, denoted π_i, can be interpreted as the probability that all individuals in the population can eventually all trace their ancestry to i. Reproductive values can be calculated as the unique solution to the linear system

$$\sum_{j=1}^{N} e_{ij}^{\circ}\pi_j = \sum_{j=1}^{N} e_{ji}^{\circ}\pi_i, \qquad [6]$$

subject to the constraint $\sum_{i=1}^{N}\pi_i = 1$ (4, 5).

Given these weights, which are used to adjust for heterogeneity within the system, the RV-weighted frequency of C is $\hat{x} := \sum_{i=1}^{N}\pi_i x_i$. Let $\rho_C(\boldsymbol{\xi})$ be the probability that cooperators fix, given an initial configuration $\boldsymbol{\xi} \in \{0,1\}^N$. Under neutral drift, we have $\rho_C^{\circ}(\boldsymbol{\xi}) = \widehat{\boldsymbol{\xi}}$. For $\delta \geqslant 0$, we know from (5, Equation 33) that

$$\rho_C(\boldsymbol{\xi}) = \widehat{\boldsymbol{\xi}} + \sum_{t=0}^{\infty} \mathbb{E}\left[\widehat{x}(t+1) - \widehat{x}(t) \mid \mathbf{x}(0) = \boldsymbol{\xi}\right]. \qquad [7]$$

That is, $\rho_C(\boldsymbol{\xi})$ is its value under neutral drift, plus a correction term.

Letting $\widehat{\Delta}_{\mathrm{sel}}(\mathbf{x},t) := \mathbb{E}\left[\widehat{x}(t+1) - \widehat{x}(t) \mid \mathbf{x}(t) = \mathbf{x}\right]$, we see that

$$\begin{aligned}\rho_C(\boldsymbol{\xi}) &= \widehat{\boldsymbol{\xi}} + \sum_{t=0}^{\infty}\sum_{\mathbf{x}\in\{0,1\}^N} \mathbb{E}\left[\widehat{x}(t+1) - \widehat{x}(t) \mid \mathbf{x}(t) = \mathbf{x}\right]\mathbb{P}\left[\mathbf{x}(t) = \mathbf{x} \mid \mathbf{x}(0) = \boldsymbol{\xi}\right] \\ &= \widehat{\boldsymbol{\xi}} + \sum_{t=0}^{\infty}\mathbb{E}\left[\widehat{\Delta}_{\mathrm{sel}}(\mathbf{x}(t),t) \mid \mathbf{x}(0) = \boldsymbol{\xi}\right]. \end{aligned} \qquad [8]$$

By the definition of reproductive value, we have $\widehat{\Delta}_{\mathrm{sel}}^{\circ}(\mathbf{x},t) = 0$ for all $\mathbf{x} \in \{0,1\}^N$ and $t \geqslant 0$. This property, in fact, is the main reason RV-weighting is useful here, owing to the resulting fact that

$$\left.\frac{d}{d\delta}\right|_{\delta=0}\rho_C(\boldsymbol{\xi}) = \sum_{t=0}^{\infty}\mathbb{E}^{\circ}\left[\left.\frac{d}{d\delta}\right|_{\delta=0}\widehat{\Delta}_{\mathrm{sel}}(\mathbf{x}(t),t) \mid \mathbf{x}(0) = \boldsymbol{\xi}\right], \qquad [9]$$

which allows for the separation of trait assortment and the effects of selection. Moreover, we have (4, 5)

$$\widehat{\Delta}_{\mathrm{sel}}(\mathbf{x},t) = \sum_{i=1}^{N}\pi_i\sum_{j=1}^{N}\left(x_j - x_i\right)e_{ji}(\mathbf{x},t). \qquad [10]$$

Within this equation, only the terms $e_{ji}(\mathbf{x},t)$ depend on δ (and thus on the game). Denoting $\mathbf{x}_I := \prod_{i\in I}x_i$ for $I \subseteq \{1,\dots,N\}$, we know that there exist unique coefficients $c_I^{ij}(t)$ such that

$$\left.\frac{d}{d\delta}\right|_{\delta=0}e_{ij}(\mathbf{x},t) = \sum_{I\subseteq\{1,\dots,N\}}c_I^{ij}(t)\,\mathbf{x}_I \qquad [11]$$

for all $\mathbf{x} \in \{0,1\}^N$. Putting everything together, we find that the first-order effect of selection is

$$\left.\frac{d}{d\delta}\right|_{\delta=0}\rho_C(\boldsymbol{\xi}) = \sum_{t=0}^{\infty}\sum_{i,j=1}^{N}\pi_i\sum_{I\subseteq\{1,\dots,N\}}c_I^{ji}(t)\,\mathbb{E}^{\circ}\left[\left(x_j(t) - x_i(t)\right)\mathbf{x}_I(t) \mid \mathbf{x}(0) = \boldsymbol{\xi}\right]. \qquad [12]$$

Of the three considerations we mentioned previously, the only one remaining is the assortment of traits under neutral drift, which here is represented by $\mathbb{E}^{\circ}\left[\left(x_j(t) - x_i(t)\right)\mathbf{x}_I(t) \mid \mathbf{x}(0) = \boldsymbol{\xi}\right]$.

It turns out that, in the donation game, we need to consider subsets I in the summation above such that $|I| = 1$. The reason why is due to the degree-one dependence of payoffs on $\mathbf{x}$. In the class of models we consider here, $e_{ij}(\mathbf{x},t)$ depends on $\mathbf{x}$ only through how $\mathbf{x}$ determines fitness. In particular, we can think of e_{ij} as a function of $F(\mathbf{x}) \in \mathbb{R}^N$ directly, where $F_i(\mathbf{x},t) = e^{\delta u_i(\mathbf{x},t)}$. Therefore,

$$\left.\frac{d}{d\delta}\right|_{\delta=0}e_{ij}(\mathbf{x},t) = \sum_{k=1}^{N}m_k^{ij}u_k(\mathbf{x},t), \qquad [13]$$

where m_k^{ij} are the partial derivatives of e_{ij} with respect to fitness, evaluated at neutral drift (10). Since $u_k(\mathbf{x},t)$ is a degree-one function of $\mathbf{x}$ in the donation game, and since the coefficients $c_I^{ij}(t)$ are unique, it follows that only those subsets I of size one in Eq. 11 are needed. Thus,

$$\left.\frac{d}{d\delta}\right|_{\delta=0}\rho_C(\boldsymbol{\xi}) = \sum_{i,j,k=1}^{N}\pi_i\sum_{t=0}^{\infty}c_k^{ji}(t)\,\mathbb{E}^{\circ}\left[\left(x_j(t)-x_i(t)\right)x_k(t)\mid\mathbf{x}(0)=\boldsymbol{\xi}\right]. \quad [14]$$

If the terms $c_k^{ji}(t)$ do not depend on t, as is the case with static interaction networks, then evaluating the terms $\sum_{t=0}^{\infty}\mathbb{E}^{\circ}\left[\left(x_j(t)-x_i(t)\right)x_k(t)\mid\mathbf{x}(0)=\boldsymbol{\xi}\right]$ can be accomplished by solving a system of equations (5). The temporal heterogeneity introduced by the dependence of $c_k^{ji}(t)$ on t complicates this problem, which marks an important theoretical departure from earlier studies. We now turn to a specific and widely studied update rule to explore the evaluation of Eq. 14 in more depth.

S3. Temporal interaction networks and death-Birth updating

Selection condition. Under death-Birth updating, an individual is chosen uniformly-at-random from the population for death. The neighbors of this individual on the replacement network then compete, with probability proportional to fitness, to reproduce and have the offspring fill the vacancy. The terms “death” and “reproduction” need not be interpreted literally within this model; in models of cultural transmission, “death” could indicate the choice of an individual to update his or her behavior, while “reproduction” could indicate the imitation of behaviors rather than birth of a new individual.

In state $\mathbf{x}$ at time t, the probability that j is replaced by the offspring of i is

$$e_{ij}(\mathbf{x},t) = \frac{1}{N}\frac{F_i(\mathbf{x},t)\,w_{ij}}{\sum_{k=1}^{N}F_k(\mathbf{x},t)\,w_{kj}}. \quad [15]$$

Under neutral drift, we have $e_{ij}^{\circ} = p_{ji}/N$, and reproductive value is well-known to be $\pi_i = w_i/W$ (1). Using the fact that $F_i(\mathbf{x},t) = e^{\delta u_i(\mathbf{x},t)}$, we see from Eqs. 3 and 15 that

$$\begin{aligned}\left.\frac{d}{d\delta}\right|_{\delta=0}e_{ij}(\mathbf{x},t) &= \frac{1}{N}p_{ji}\left(u_i(\mathbf{x},t)-\sum_{k=1}^{N}p_{jk}u_k(\mathbf{x},t)\right)\\ &= \frac{1}{N}p_{ji}\left(-cq_i(t)\,x_i+b\sum_{\ell=1}^{N}q_{i\ell}(t)\,x_\ell-\sum_{k=1}^{N}p_{jk}\left(-cq_k(t)\,x_k+b\sum_{\ell=1}^{N}q_{k\ell}(t)\,x_\ell\right)\right).\end{aligned} \quad [16]$$

Therefore, combining this expression with Eqs. 9 and 10, we obtain

$$\begin{aligned}\left.\frac{d}{d\delta}\right|_{\delta=0}\rho_C(\boldsymbol{\xi}) &= \frac{c}{N}\Bigg(-\sum_{t=0}^{\infty}\sum_{i=1}^{N}\pi_i q_i(t)\,\mathbb{E}^{\circ}\left[x_i(t)\mid\mathbf{x}(0)=\boldsymbol{\xi}\right]\\ &\qquad+\sum_{t=0}^{\infty}\sum_{i,j=1}^{N}\pi_i p_{ij}^{(2)}q_j(t)\,\mathbb{E}^{\circ}\left[x_i(t)\,x_j(t)\mid\mathbf{x}(0)=\boldsymbol{\xi}\right]\Bigg)\\ &\quad-\frac{b}{N}\Bigg(-\sum_{t=0}^{\infty}\sum_{i,j=1}^{N}\pi_i q_{ij}(t)\,\mathbb{E}^{\circ}\left[x_i(t)\,x_j(t)\mid\mathbf{x}(0)=\boldsymbol{\xi}\right]\\ &\qquad+\sum_{t=0}^{\infty}\sum_{i,j,k=1}^{N}\pi_i p_{ij}^{(2)}q_{jk}(t)\,\mathbb{E}^{\circ}\left[x_i(t)\,x_k(t)\mid\mathbf{x}(0)=\boldsymbol{\xi}\right]\Bigg)\\ &= \frac{c}{N}\Bigg(\sum_{t=0}^{\infty}\sum_{i=1}^{N}\pi_i q_i(t)\left(\frac{1}{N}-\mathbb{E}^{\circ}\left[x_i(t)\mid\mathbf{x}(0)=\boldsymbol{\xi}\right]\right)\\ &\qquad-\sum_{t=0}^{\infty}\sum_{i,j=1}^{N}\pi_i p_{ij}^{(2)}q_j(t)\left(\frac{1}{N}-\mathbb{E}^{\circ}\left[x_i(t)\,x_j(t)\mid\mathbf{x}(0)=\boldsymbol{\xi}\right]\right)\Bigg)\\ &\quad+\frac{b}{N}\Bigg(-\sum_{t=0}^{\infty}\sum_{i,j=1}^{N}\pi_i q_{ij}(t)\left(\frac{1}{N}-\mathbb{E}^{\circ}\left[x_i(t)\,x_j(t)\mid\mathbf{x}(0)=\boldsymbol{\xi}\right]\right)\\ &\qquad+\sum_{t=0}^{\infty}\sum_{i,j,k=1}^{N}\pi_i p_{ij}^{(2)}q_{jk}(t)\left(\frac{1}{N}-\mathbb{E}^{\circ}\left[x_i(t)\,x_k(t)\mid\mathbf{x}(0)=\boldsymbol{\xi}\right]\right)\Bigg).\end{aligned} \quad [17]$$

Since individuals are chosen for death uniformly-at-random in this model, it is natural that new mutants appear uniformly within the population. So, for the sake of initial conditions, we consider not a deterministic configuration $\boldsymbol{\xi}\in\{0,1\}^N$, but a distribution μ_C^{unif} over

configurations such that

$$\mu_C^{\mathrm{unif}}\left(\boldsymbol{\xi}\right)=\begin{cases}\dfrac{1}{N} & \displaystyle\sum_{i=1}^{N}\xi_i=1,\\ 0 & \text{otherwise.}\end{cases} \tag{18}$$

Under this distribution, we can apply duality between coalescence and the neutral case of the model (11) to rewrite the expectations appearing in Eq. 17. Consider the ancestral process going backward in time, defined by the neutral replacement rule. Any subset of nodes will eventually descend from a common ancestor, and we let T^{coal} be the number of steps backward until this happens for the first time. Given starting nodes of i and j, we denote by $\mathbb{P}_{(i,j)}\left[T^{\mathrm{coal}}=n\right]$ the probability that it takes exactly n steps for i and j to descend from a common ancestor in the resulting coalescing random walk. Based on Eq. 15, these terms can be calculated using the recurrence

$$\mathbb{P}_{(i,j)}\left[T^{\mathrm{coal}}=n\right]=\begin{cases}\sum_{k=1}^{N}\frac{p_{ik}}{N}\mathbb{P}_{(k,j)}\left[T^{\mathrm{coal}}=n-1\right]+\sum_{k=1}^{N}\frac{p_{jk}}{N}\mathbb{P}_{(i,k)}\left[T^{\mathrm{coal}}=n-1\right]+\frac{N-2}{N}\mathbb{P}_{(i,j)}\left[T^{\mathrm{coal}}=n-1\right], & i\neq j\\ 0, & i=j\end{cases} \tag{19}$$

when $n>0$. When $n=0$, we have $\mathbb{P}_{(i,j)}\left[T^{\mathrm{coal}}=0\right]=1$ if $i=j$ and 0 otherwise. Moreover, exploiting the backward-forward duality in the neutral process, we find that

$$\begin{aligned}\mathbb{E}^{\circ}\left[\frac{1}{N}-x_i\left(t\right)x_j\left(t\right)\mid\mathbf{x}\left(0\right)\sim\mu_C\right]&=\frac{1}{N}-\mathbb{P}^{\circ}\left[x_i\left(t\right)=x_j\left(t\right)=1\mid\mathbf{x}\left(0\right)\sim\mu_C\right]\\&=\frac{1}{N}-\frac{1}{N}\mathbb{P}_{(i,j)}\left[T^{\mathrm{coal}}\leqslant t\right]\\&=\frac{1}{N}\mathbb{P}_{(i,j)}\left[T^{\mathrm{coal}}>t\right].\end{aligned} \tag{20}$$

Moreover, if the lineages leading to i and j to coalesce exactly n time steps into the past, then we can count how many time steps involve updates (i.e., death) in at least one of these lineages prior to coalescence. In each time step, an update occurs in at least one of these lineages if and only if it occurs in exactly one of these lineages, by the definition of death-Birth updating, which happens with probability $2/N$. In particular, there is an embedded chain involving m time steps, corresponding to exactly those time steps in which exactly one of these lineages is updated. Since coalescence happens at time n and not before, it must be true that one of these two lineages is chosen for an update exactly $n-1$ steps into the past. The remaining $m-1$ steps can be arranged in any order along $n-1$ update steps in the death-Birth process, so the probability of an embedded length of exactly m prior to coalescence n steps into the past is $\left(2/N\right)\binom{n-1}{m-1}\left(2/N\right)^{m-1}\left(1-2/N\right)^{n-m}$.

Within each embedded step, the probability that the update occurs in the lineage leading to i is $1/2$; otherwise, it occurs in the lineage leading to j. Let τ be the number of steps required for two random walks on the replacement network to coalesce, and let $\mathbb{P}_{(i,j)}\left[\tau=m\right]$ be the probability that these two random walks coalesce in exactly m steps, given starting locations of i and j. For $m>0$,

$$\mathbb{P}_{(i,j)}\left[\tau=m\right]=\begin{cases}\dfrac{1}{2}\displaystyle\sum_{k=1}^{N}p_{ik}\mathbb{P}_{(k,j)}\left[\tau=m-1\right]+\dfrac{1}{2}\displaystyle\sum_{k=1}^{N}p_{jk}\mathbb{P}_{(i,k)}\left[\tau=m-1\right] & i\neq j\\ 0 & i=j.\end{cases} \tag{21}$$

When $m=0$, we have $\mathbb{P}_{(i,j)}\left[\tau=0\right]=1$ if $i=j$ and 0 otherwise. From these terms, we have

$$\mathbb{P}_{(i,j)}\left[T^{\mathrm{coal}}=n\right]=\frac{2}{N}\sum_{m=1}^{n}\binom{n-1}{m-1}\left(\frac{2}{N}\right)^{m-1}\left(1-\frac{2}{N}\right)^{n-m}\mathbb{P}_{(i,j)}\left[\tau=m\right], \tag{22}$$

which (in principle) allows the coalescence times to be calculated from the embedded random walk.

Therefore, if we write ρ_C for $\mathbb{E}_{\boldsymbol{\xi}\sim\mu_C}\left[\rho_C\left(\boldsymbol{\xi}\right)\right]$, then $\rho_C=1/N+\delta\left.\frac{d}{d\delta}\right|_{\delta=0}\rho_C+O\left(\delta^2\right)$, where

$$\begin{aligned}\left.\frac{d}{d\delta}\right|_{\delta=0}\rho_C=&-\frac{c}{N^2}\underbrace{\sum_{t=0}^{\infty}\sum_{i,j=1}^{N}\pi_i p_{ij}^{(2)}q_j\left(t\right)\mathbb{P}_{(i,j)}\left[T^{\mathrm{coal}}>t\right]}_{\text{T20 in Algorithm 1}}\\&+\frac{b}{N^2}\left(\underbrace{\sum_{t=0}^{\infty}\sum_{i,j,k=1}^{N}\pi_i p_{ij}^{(2)}q_{jk}\left(t\right)\mathbb{P}_{(i,k)}\left[T^{\mathrm{coal}}>t\right]}_{\text{T21 in Algorithm 1}}\right.\\&\left.-\underbrace{\sum_{t=0}^{\infty}\sum_{i,j=1}^{N}\pi_i q_{ij}\left(t\right)\mathbb{P}_{(i,j)}\left[T^{\mathrm{coal}}>t\right]}_{\text{T01 in Algorithm 1}}\right).\end{aligned} \tag{23}$$

According to Eq. 19, we can obtain the recurrence of $\mathbb{P}_{(i,j)}\left[T^{\text{coal}} > n\right]$ by summing both side of this equation from $\mathbb{P}_{(i,j)}\left[T^{\text{coal}} = n+1\right]$ to infinity

$$\mathbb{P}_{(i,j)}\left[T^{\text{coal}} > n\right] = \begin{cases} \sum_{k=1}^{N} \frac{p_{ik}}{N} \mathbb{P}_{(k,j)}\left[T^{\text{coal}} > n-1\right] + \sum_{k=1}^{N} \frac{p_{jk}}{N} \mathbb{P}_{(i,k)}\left[T^{\text{coal}} > n-1\right] & \\ + \frac{N-2}{N} \mathbb{P}_{(i,j)}\left[T^{\text{coal}} > n-1\right], & i \neq j \\ 0, & i = j \end{cases} \quad [24]$$

for $n > 0$. And $\mathbb{P}_{(i,j)}\left[T^{\text{coal}} > 0\right] = 0$ if $i = j$ and 1 otherwise. To efficiently compute the critical ratio $(\frac{b}{c})^*$ by letting $\rho_C > \frac{1}{N}$ ($\left.\frac{d}{d\delta}\right|_{\delta=0} \rho_C > 0$ in Eq. 23), we take a matrix M^P to denote the coalescent probability between any pair of nodes at each step of the iteration where $M^P_{ij} = \mathbb{P}_{(i,j)}\left[T^{\text{coal}} > t\right]$, the detailed implementation is shown in Algorithm 1. With this computational procedure, only the adjacent matrices of temporal interactions and the corresponding replacement network are required to obtain the critical benefit-to-cost ratio $(\frac{b}{c})^*$.

Algorithm 1 Calculation of the critical ratio $(\frac{b}{c})^*$.

Require: Adjacent matrix of temporal interactions $M^I(t)$, Adjacent matrix of replacement graph M^R

Compute π_i for all i, compute p_{ij} ($p^{(2)}_{ij}$) in the matrix form $P10$ ($P20$) with M^R
Compute $q_{ij}(t)$ and $q_i(t)$ in the matrix form with $M^I(t)$
Initialize time step $t = 0$
Initialize $T20, T01, T21 = 0$
Initialize M^P with $M^P_{(i,i)} = 0$ for all i, and $M^P_{(i,j)} = 1$ for all $i \neq j$
while $\frac{1}{N(N-1)} \sum_{i,j} |M^P_{(i,j)}| > 10^{-6}$ **do**
  $T20 \leftarrow T20 + \sum_{i,j=1}^{N} \pi_i p^{(2)}_{ij} q_j(t) M^P_{(i,j)}$
  $T21 \leftarrow T21 + \sum_{i,j,k=1}^{N} \pi_i p^{(2)}_{ij} q_{jk}(t) M^P_{(i,k)}$
  $T01 \leftarrow T01 + \sum_{i,j=1}^{N} \pi_i q_{ij}(t) M^P_{(i,j)}$
  $M^P = \frac{1}{N} P10 M^P + \frac{1}{N} M^P P10' + \frac{N-2}{N} M^P$
  $M^P_{(i,i)} \leftarrow 0$ for all i
  $t \leftarrow t+1$
$\left(\frac{b}{c}\right)^* = T20/(T21 - T01)$

Evaluating the selection condition. In practice, the computational efficiency of calculating $\mathbb{P}_{(i,j)}\left[T^{\text{coal}} > t\right]$ directly (e.g., using Eq. 24) tends to decrease as the network size grows. Here, we consider the calculations involved in Eq. 23 when the duration of each snapshot is frozen over a window of time steps. Suppose that the interaction networks change at times $\{TN/2\}_{T=1}^{\infty}$, such that each interaction network persists for a duration of $N/2$ time steps. (We assume, for notational simplicity, that N is divisible by 2; otherwise, we can replace $TN/2$ by $\lfloor TN/2 \rfloor$.) Eq. 23 then becomes

$$\begin{aligned} \left.\frac{d}{d\delta}\right|_{\delta=0} \rho_C = &-\frac{c}{N^2} \sum_{T=0}^{\infty} \sum_{i,j=1}^{N} \pi_i q_j(TN/2)\, p^{(2)}_{ij} \sum_{t=TN/2}^{(T+1)N/2-1} \mathbb{P}_{(i,j)}\left[T^{\text{coal}} > t\right] \\ &+ \frac{b}{N^2} \left(\sum_{T=0}^{\infty} \sum_{i,j,k=1}^{N} \pi_i p^{(2)}_{ij} q_{jk}(TN/2) \sum_{t=TN/2}^{(T+1)N/2-1} \mathbb{P}_{(i,k)}\left[T^{\text{coal}} > t\right] \right. \\ &\left. - \sum_{T=0}^{\infty} \sum_{i,j=1}^{N} \pi_i q_{ij}(TN/2) \sum_{t=TN/2}^{(T+1)N/2-1} \mathbb{P}_{(i,j)}\left[T^{\text{coal}} > t\right] \right). \end{aligned} \quad [25]$$

The challenge, then, is to calculate the terms $\sum_{t=TN/2}^{(T+1)N/2-1} \mathbb{P}_{(i,j)}\left[T^{\text{coal}} > t\right]$. After making the approximation

$$\mathbb{P}_{(i,j)}\left[T^{\text{coal}} > t\right] \approx \mathbb{P}_{(i,j)}\left[\tau > \lfloor 2t/N \rfloor\right],$$

where the accuracy is shown in Fig. S15, we further obtain that

$$\sum_{t=TN/2}^{(T+1)N/2-1} \mathbb{P}_{(i,j)}\left[T^{\text{coal}} > t\right] \approx \frac{N}{2} \mathbb{P}_{(i,j)}\left[\tau > T\right].$$

Using this approximation, we can rewrite Eq. 23 as

$$\left.\frac{d}{d\delta}\right|_{\delta=0} \rho_C \approx -\frac{c}{2N} \tau^{(2,0)} + \frac{b}{2N} \left(\tau^{(2,1)} - \tau^{(0,1)}\right), \quad [26]$$

where

$$\tau^{(2,0)} = \sum_{T=0}^{\infty} \sum_{i,j=1}^{N} \pi_i p_{ij}^{(2)} q_j \left(TN/2\right) \mathbb{P}_{(i,j)} \left[\tau > T\right]; \quad [27a]$$

$$\tau^{(2,1)} = \sum_{T=0}^{\infty} \sum_{i,j,k=1}^{N} \pi_i p_{ij}^{(2)} q_{jk} \left(TN/2\right) \mathbb{P}_{(i,k)} \left[\tau > T\right]; \quad [27b]$$

$$\tau^{(0,1)} = \sum_{T=0}^{\infty} \sum_{i,j=1}^{N} \pi_i q_{ij} \left(TN/2\right) \mathbb{P}_{(i,j)} \left[\tau > T\right]. \quad [27c]$$

According to Eq. 26, cooperation is favored over defection whenever the benefit-to-cost ratio of cooperation, b/c, exceeds the critical threshold

$$\left(\frac{b}{c}\right)^{*} \coloneqq \frac{\tau^{(2,0)}}{\tau^{(2,1)} - \tau^{(0,1)}}. \quad [28]$$

Mean-field approximation. In what follows, we let $\widetilde{q}_{ij}(T) \coloneqq q_{ij}(TN/2)$ and $\widetilde{q}_i(T) \coloneqq q_i(TN/2)$. For simplification, we use $\mathbb{P}_{(i,j)}(\tau)$ to denote the probability that two random walks coalesce in exactly τ steps, given starting locations of i and j with each one taking a step with probability $1/2$ at each point in time. Then, we have $\mathbb{P}_{(i,j)}\left[\tau > T\right] = \sum_{\tau=T+1}^{\infty} \mathbb{P}_{(i,j)}(\tau)$ in Eq. 27. By Eq. 27, we have

$$\tau^{(2,0)} = \sum_{\tau=1}^{\infty} \sum_{i,j=1}^{N} \pi_i p_{ij}^{(2)} Q_j(\tau) \mathbb{P}_{(i,j)}(\tau); \quad [29a]$$

$$\tau^{(2,1)} = \sum_{\tau=1}^{\infty} \sum_{i,j,k=1}^{N} \pi_i p_{ij}^{(2)} Q_{jk}(\tau) \mathbb{P}_{(i,k)}(\tau); \quad [29b]$$

$$\tau^{(0,1)} = \sum_{\tau=1}^{\infty} \sum_{i,j=1}^{N} \pi_i Q_{ij}(\tau) \mathbb{P}_{(i,j)}(\tau), \quad [29c]$$

where $Q_{ij}(\tau) = \sum_{T=0}^{\tau-1} \widetilde{q}_{ij}(T)$ and $Q_i(\tau) = \sum_j Q_{ij}(\tau) = \sum_{T=0}^{\tau-1} \widetilde{q}_i(T)$.

Applying the recurrence relation in Eq. 21 we have

$$\begin{aligned}
\tau^{(0,1)} = {} & \frac{1}{4} \sum_{\tau=1}^{\infty} \left(\sum_{i,j,k=1}^{N} \pi_i p_{ik}^{(2)} Q_{kj}(\tau) \mathbb{P}_{(i,j)}(\tau) + 2 \sum_{i,j,k,m=1}^{N} \pi_i p_{ik} Q_{km}(\tau) p_{mj} \mathbb{P}_{(i,j)}(\tau) + \sum_{i,j,k=1}^{N} \pi_i Q_{ik}(\tau) p_{kj}^{(2)} \mathbb{P}_{(i,j)}(\tau) \right) \\
& + \frac{1}{4} \sum_{\tau=0}^{\infty} \left(\sum_{i,j,k=1}^{N} \pi_i p_{ik}^{(2)} \widetilde{q}_{kj}(\tau) \mathbb{P}_{(i,j)}(\tau) + 2 \sum_{i,j,k,m=1}^{N} \pi_i p_{ik} \widetilde{q}_{km}(\tau) p_{mj} \mathbb{P}_{(i,j)}(\tau) + \sum_{i,j,k=1}^{N} \pi_i \widetilde{q}_{ik}(\tau) p_{kj}^{(2)} \mathbb{P}_{(i,j)}(\tau) \right) \\
& - \frac{1}{2} \sum_{\tau=1}^{\infty} \sum_{i,k,m=1}^{N} \pi_i \left(p_{ik} Q_{ki}(\tau) + Q_{ik}(\tau) p_{ki} \right) p_{im} \mathbb{P}_{(i,m)}(\tau) \\
& - \frac{1}{2} \sum_{\tau=0}^{\infty} \sum_{i,k,m=1}^{N} \pi_i \left(p_{ik} \widetilde{q}_{ki}(\tau) + \widetilde{q}_{ik}(\tau) p_{ki} \right) p_{im} \mathbb{P}_{(i,m)}(\tau) \\
& + \frac{1}{2} \sum_{\tau=0}^{\infty} \sum_{i,j,k=1}^{N} \pi_i \left(p_{ik} \widetilde{q}_{kj}(\tau) + \widetilde{q}_{ik}(\tau) p_{kj} \right) \mathbb{P}_{(i,j)}(\tau).
\end{aligned} \quad [30]$$

Then, $\tau^{(2,1)} - \tau^{(0,1)}$ is given by

$$\begin{aligned}
\tau^{(2,1)} - \tau^{(0,1)} = \frac{1}{4}\sum_{\tau=1}^{\infty}&\left(\sum_{i,j,k=1}^{N}\pi_i 3p_{ik}^{(2)}Q_{kj}(\tau)\mathbb{P}_{(i,j)}(\tau) - 2\sum_{i,j,k,m=1}^{N}\pi_i p_{ik}Q_{km}(\tau)p_{mj}\mathbb{P}_{(i,j)}(\tau)\right.\\
&\left.-\sum_{i,j,k=1}^{N}\pi_i Q_{ik}(\tau)p_{kj}^{(2)}\mathbb{P}_{(i,j)}(\tau)\right) - \frac{1}{4}\sum_{\tau=0}^{\infty}\left(\sum_{i,j,k=1}^{N}\pi_i p_{ik}^{(2)}\widetilde{q}_{kj}(\tau)\mathbb{P}_{(i,j)}(\tau)\right.\\
&\left.+2\sum_{i,j,k,m=1}^{N}\pi_i p_{ik}\widetilde{q}_{km}(\tau)p_{mj}\mathbb{P}_{(i,j)}(\tau) + \sum_{i,j,k=1}^{N}\pi_i\widetilde{q}_{ik}(\tau)p_{kj}^{(2)}\mathbb{P}_{(i,j)}(\tau)\right)\\
&+\frac{1}{2}\sum_{\tau=1}^{\infty}\sum_{i,k,m=1}^{N}\pi_i\left(p_{ik}Q_{ki}(\tau) + Q_{ik}(\tau)p_{ki}\right)p_{im}\mathbb{P}_{(i,m)}(\tau)\\
&+\frac{1}{2}\sum_{\tau=0}^{\infty}\sum_{i,k,m=1}^{N}\pi_i\left(p_{ik}\widetilde{q}_{ki}(\tau) + \widetilde{q}_{ik}(\tau)p_{ki}\right)p_{im}\mathbb{P}_{(i,m)}(\tau)\\
&-\frac{1}{2}\sum_{\tau=0}^{\infty}\sum_{i,j,k=1}^{N}\pi_i\left(p_{ik}\widetilde{q}_{kj}(\tau) + \widetilde{q}_{ik}(\tau)p_{kj}\right)\mathbb{P}_{(i,j)}(\tau).
\end{aligned} \qquad [31]$$

Applying the mean-field approximation, we replace each $\mathbb{P}_{(i,j)}(\tau)$ by $\mathbb{P}(\tau)$ when $i \neq j$ and $\tau \geq 1$, where $\mathbb{P}(\tau)$ is the average probability over any pair of different nodes. Then, the $\tau^{(2,1)} - \tau^{(0,1)}$ is approximated by

$$\begin{aligned}
\tau^{(2,1)} - \tau^{(0,1)} \approx\ &\frac{1}{2}\sum_{\tau=1}^{\infty}\sum_{i,j=1}^{N}\pi_i\left(p_{ij}Q_{ji}(\tau) + Q_{ij}(\tau)p_{ji}\right)\mathbb{P}(\tau) + \frac{1}{2}\sum_{\tau=0}^{\infty}\sum_{i,j=1}^{N}\pi_i\left(p_{ij}\widetilde{q}_{ji}(\tau) + \widetilde{q}_{ij}(\tau)p_{ji}\right)\mathbb{P}(\tau)\\
&-2\sum_{\tau=0}^{\infty}\sum_{i=1}^{N}\pi_i\widetilde{q}_i(\tau)\mathbb{P}(\tau) + \varepsilon_b.
\end{aligned} \qquad [32]$$

where

$$\begin{aligned}
\varepsilon_b = -\frac{1}{4}\sum_{\tau=1}^{\infty}&\left(3\sum_{i,k=1}^{N}\pi_i p_{ik}^{(2)}Q_{ki}(\tau)\mathbb{P}(\tau) - 2\sum_{i,k,m=1}^{N}\pi_i p_{ik}Q_{km}(\tau)p_{mi}\mathbb{P}(\tau) - \sum_{i,k=1}^{N}\pi_i Q_{ik}(\tau)p_{ki}^{(2)}\mathbb{P}(\tau)\right)\\
+\frac{1}{4}\sum_{\tau=1}^{\infty}&\left(\sum_{i,k=1}^{N}\pi_i p_{ik}^{(2)}\widetilde{q}_{ki}(\tau)\mathbb{P}(\tau) + 2\sum_{i,k,m=1}^{N}\pi_i p_{ik}\widetilde{q}_{km}(\tau)p_{mi}\mathbb{P}(\tau) + \sum_{i,k=1}^{N}\pi_i\widetilde{q}_{ik}(\tau)p_{ki}^{(2)}\mathbb{P}(\tau)\right)\\
-\frac{1}{4}&\left(\sum_{i,k=1}^{N}\pi_i p_{ik}^{(2)}\widetilde{q}_{ki}(0) + 2\sum_{i,k,m=1}^{N}\pi_i p_{ik}\widetilde{q}_{km}(0)p_{mi} + \sum_{i,k=1}^{N}\pi_i\widetilde{q}_{ik}(0)p_{ki}^{(2)}\right)\\
+\frac{1}{2}\sum_{\tau=1}^{\infty}&\sum_{i,k=1}^{N}\pi_i\left(p_{ik}\widetilde{q}_{ki}(\tau) + \widetilde{q}_{ik}(\tau)p_{ki}\right)\mathbb{P}(\tau) - \frac{1}{2}\sum_{i,k=1}^{N}\pi_i\left(p_{ik}\widetilde{q}_{ki}(0) + \widetilde{q}_{ik}(0)p_{ki}\right).
\end{aligned}$$

Similarly, we have

$$\begin{aligned}
\tau^{(2,0)} &= \tau^{(2,0)} - \tau^{(1,0)} + \tau^{(1,0)}\\
&= \frac{1}{2}\sum_{\tau=1}^{\infty}\sum_{i,j=1}^{N}\pi_i p_{ij}Q_j(\tau)\mathbb{P}_{(i,j)}(\tau) + \frac{1}{2}\sum_{\tau=1}^{\infty}\left(\sum_{i,j=1}^{N}\pi_i p_{ij}^{(2)}Q_j(\tau)\mathbb{P}_{(i,j)}(\tau) - \sum_{i,j,k=1}^{N}\pi_i p_{ik}Q_k(\tau)p_{kj}\mathbb{P}_{(i,j)}(\tau)\right)\\
&-\frac{1}{2}\sum_{\tau=0}^{\infty}\left(\sum_{i,j=1}^{N}\pi_i p_{ij}^{(2)}\widetilde{q}_j(\tau)\mathbb{P}_{(i,j)}(\tau) + \sum_{i,j,k=1}^{N}\pi_i p_{ik}\widetilde{q}_k(\tau)p_{kj}\mathbb{P}_{(i,j)}(\tau)\right).
\end{aligned}$$

By replacing each $\mathbb{P}_{(i,j)}(\tau)$ with $\mathbb{P}(\tau)$ when $i \neq j$ and $\tau \geq 1$, we have

$$\tau^{(2,0)} \approx \sum_{\tau=1}^{\infty}\sum_{i=1}^{N}\pi_i Q_i(\tau)\mathbb{P}(\tau) + \varepsilon_c, \qquad [33]$$

where

$$\varepsilon_c = -\frac{1}{2}\sum_{\tau=0}^{\infty}\sum_{i,j=1}^{N}\pi_i p_{ij}^{(2)}\widetilde{q}_j(\tau)\mathbb{P}(\tau) - \frac{1}{2}\sum_{\tau=0}^{\infty}\sum_{i,j,k=1}^{N}\pi_i p_{ik}\widetilde{q}_k(\tau)p_{kj}\mathbb{P}(\tau) - \frac{1}{2}\sum_{\tau=1}^{\infty}\sum_{i=1}^{N}\pi_i p_{ii}^{(2)}Q_i(\tau)\mathbb{P}(\tau)$$
$$+\frac{1}{2}\sum_{\tau=1}^{\infty}\sum_{i,k=1}^{N}\pi_i p_{ik}Q_k(\tau)p_{ki}\mathbb{P}(\tau) + \frac{1}{2}\sum_{\tau=1}^{\infty}\sum_{i=1}^{N}\pi_i p_{ii}^{(2)}\widetilde{q}_i(\tau)\mathbb{P}(\tau)$$
$$+\frac{1}{2}\sum_{\tau=1}^{\infty}\sum_{i,k=1}^{N}\pi_i p_{ik}\widetilde{q}_k(\tau)p_{ki}\mathbb{P}(\tau) - \frac{1}{2}\sum_{i=1}^{N}\pi_i p_{ii}^{(2)}\widetilde{q}_i(0) - \frac{1}{2}\sum_{i,k=1}^{N}\pi_i p_{ik}\widetilde{q}_k(0)p_{ki}.$$

For static interactions, we have

$$\tau^{(2,1)} - \tau^{(0,1)} \approx (\overline{\tau}+1)\frac{1}{2}\sum_{i,j=1}^{N}\pi_i\left(p_{ij}q_{ji} + q_{ij}p_{ji}\right) - 2\sum_{i=1}^{N}\pi_i q_i + \epsilon_b,$$

where $\epsilon_b = -\frac{1}{4}\overline{\tau}\left(3\sum_{i,k=1}^{N}\pi_i p_{ik}^{(2)}q_{ki} - \sum_{i,k=1}^{N}\pi_i q_{ik}p_{ki}^{(2)} - 2\sum_{i,j,k=1}^{N}\pi_i p_{ij}q_{jk}p_{ki}\right)$. And

$$\tau^{(2,0)} \approx \overline{\tau}\sum_{i=1}^{N}\pi_i q_i + \epsilon_c,$$

where $\epsilon_c = -\frac{1}{2}\left(\sum_{i,j=1}^{N}\pi_i p_{ij}^{(2)}q_j + \sum_{i,j,k=1}^{N}\pi_i p_{ik}q_k p_{kj}\right) - \frac{1}{2}\overline{\tau}\left(\sum_{i=1}^{N}\pi_i p_{ii}^{(2)}q_i - \sum_{i,j=1}^{N}\pi_i p_{ij}q_j p_{ji}\right)$.

Particularly, for static networks that the interaction coincides with replacement, our approximation results in

$$\tau^{(2,0)} \approx \overline{\tau} - 1,$$
$$\tau^{(2,1)} - \tau^{(0,1)} \approx \sum_{i=1}^{N}\pi_i p_{ii}^{(2)}\left(\overline{\tau}+1\right) - 2,$$

where $\overline{\tau} = \sum_{\tau=1}^{\infty}\tau\mathbb{P}(\tau)$ represents the average mean coalescence time between any pair of different nodes.

Temporal interactions on random regular networks. Here we explore the evolution of cooperation over temporal interactions on random regular networks, where each individual on the underlying replacement networks has equal number of neighbors, namely $w_i = k$ for each i. By applying the mean-field approximation shown in Eqs. 32 and 33, we have

$$\tau^{(2,0)} \approx \sum_{\tau=1}^{\infty}\sum_{t=0}^{\tau-1}\frac{n_A(t)}{N}\mathbb{P}(\tau) - \sum_{\tau=0}^{\infty}\frac{n_A(t)}{N}\mathbb{P}(\tau) \quad [34]$$

and

$$\tau^{(2,1)} - \tau^{(0,1)} \approx \frac{1}{k}\sum_{\tau=1}^{\infty}\sum_{t=0}^{\tau}\frac{n_A(t)}{N}\mathbb{P}(\tau) - 2\sum_{\tau=0}^{\infty}\frac{n_A(t)}{N}\mathbb{P}(\tau), \quad [35]$$

where $n_A(t)$ indicates the number of activated nodes at time t. Since $\sum_{\tau=1}^{\infty}\sum_{t=0}^{\tau}\frac{n_A(t)}{N}\mathbb{P}(\tau) \sim O(\overline{\tau})$ and $\sum_{\tau=0}^{\infty}\frac{n_A(t)}{N}\mathbb{P}(\tau) \sim O(1)$. For large networks we have the average coalescence time $\overline{\tau} \gg 1$, then we can obtain that

$$(b/c)^* = \frac{\tau^{(2,0)}}{\tau^{(2,1)} - \tau^{(0,1)}} \approx k$$

for temporal random regular networks.

S4. Other update rules

A. Imitation (IM) updating. Under imitation (IM) updating, an individual is chosen uniformly-at-random to update, and imitate the strategy from its neighbors or keep its own strategy with probability proportional to the corresponding fitness. In state $\mathbf{x}$ at time t, the probability that j is replaced by the offspring of i is

$$e_{ij}(\mathbf{x},t) = \frac{1}{N}\frac{F_i(\mathbf{x},t)\,w_{ij}^{\mathrm{IM}}}{\sum_{k=1}^{N}F_k(\mathbf{x},t)\,w_{kj}^{\mathrm{IM}}}, \quad [36]$$

where we define

$$w_{ij}^{\mathrm{IM}} = \begin{cases} w_{ij} & i \neq j \\ 1 & i = j \end{cases} \quad [37]$$

to enable each individual i keep its own strategy with probability proportional to its fitness $F_i(\mathbf{x}, t)$. Following the Eq. 9, we have

$$\begin{aligned}\left.\frac{d}{d\delta}\right|_{\delta=0}\rho_C = \frac{c}{N}\Bigg(\sum_{t=0}^{\infty}\sum_{i=1}^{N}\pi_i q_i(t)\left(\frac{1}{N} - \mathbb{E}^{\circ}\left[x_i(t) \mid \mathbf{x}(0) \sim \mu_C\right]\right) \\ - \sum_{t=0}^{\infty}\sum_{i,j=1}^{N}\pi_i p_{ij}^{\mathrm{IM}(2)} q_j(t)\left(\frac{1}{N} - \mathbb{E}^{\circ}\left[x_i(t)x_j(t) \mid \mathbf{x}(0) \sim \mu_C\right]\right)\Bigg) \\ + \frac{b}{N}\Bigg(-\sum_{t=0}^{\infty}\sum_{i,j=1}^{N}\pi_i q_{ij}(t)\left(\frac{1}{N} - \mathbb{E}^{\circ}\left[x_i(t)x_j(t) \mid \mathbf{x}(0) \sim \mu_C\right]\right) \\ + \sum_{t=0}^{\infty}\sum_{i,j,k=1}^{N}\pi_i p_{ij}^{\mathrm{IM}(2)} q_{jk}(t)\left(\frac{1}{N} - \mathbb{E}^{\circ}\left[x_i(t)x_k(t) \mid \mathbf{x}(0) \sim \mu_C\right]\right)\Bigg),\end{aligned} \quad [38]$$

where $p_{ij}^{\mathrm{IM}} = w_{ij}^{\mathrm{IM}}/(w_i + 1)$ indicates the probability of the single step random walk from i to j on the modified graph for imitation updating by adding a self-loop for each node. And the probability for two-step random walk is defined by $p_{ij}^{\mathrm{IM}(2)}$ correspondingly.

We now offer the general equation capturing the coalescent process under any update rules. We have $\mathbb{E}^{\circ}\left[x_i(t)x_j(t) \mid \mathbf{x}(0) \sim \mu_C\right]$ equals to the probability $\mathbb{P}^{\circ}\left[x_i(t) = x_j(t) = 1\right]$, which is given by the recurrence relationship

$$\begin{aligned}\mathbb{P}^{\circ}\left[x_i(t) = x_j(t) = 1\right] = \sum_{k=1}^{N} e_{ki}^{\circ}\mathbb{P}^{\circ}\left[x_k(t-1) = x_j(t-1) = 1\right] + \sum_{k=1}^{N} e_{kj}^{\circ}\mathbb{P}^{\circ}\left[x_k(t-1) = x_i(t-1) = 1\right] \\ + \sum_{k,l=1,l\notin\{i,j\}}^{N} e_{kl}^{\circ}\mathbb{P}^{\circ}\left[x_i(t-1) = x_j(t-1) = 1\right].\end{aligned} \quad [39]$$

When $t = 0$, we have $\mathbb{P}^{\circ}\left[x_i(0) = x_j(0) = 1\right] = 0$ if $i \neq j$, and $\mathbb{P}^{\circ}\left[x_i(0) = 1\right] = \frac{1}{N}$. The above equation captures the coalescent process of strategies over time in neutral case.

For imitation updating, we have

$$\begin{aligned}\mathbb{P}^{\circ}\left[x_i(t) = x_j(t) = 1\right] = \frac{1}{N}\sum_{k=1}^{N} p_{ik}^{\mathrm{IM}}\mathbb{P}^{\circ}\left[x_k(t-1) = x_j(t-1) = 1\right] + \frac{1}{N}\sum_{k=1}^{N} p_{jk}^{\mathrm{IM}}\mathbb{P}^{\circ}\left[x_k(t-1) = x_i(t-1) = 1\right] \\ + \frac{N-2}{N}\mathbb{P}^{\circ}\left[x_i(t-1) = x_j(t-1) = 1\right].\end{aligned} \quad [40]$$

Due to the backward-forward duality shown in Eq. 20, we have

$$\mathbb{P}_{(i,j)}\left[T^{\mathrm{coal}} > t\right] = \begin{cases} \frac{1}{N}\sum_{k=1}^{N} p_{ik}^{\mathrm{IM}}\mathbb{P}_{(k,j)}\left[T^{\mathrm{coal}} > t-1\right] + \frac{1}{N}\sum_{k=1}^{N} p_{jk}^{\mathrm{IM}}\mathbb{P}_{(k,i)}\left[T^{\mathrm{coal}} > t-1\right] & \\ +\frac{N-2}{N}\mathbb{P}_{(i,j)}\left[T^{\mathrm{coal}} > t-1\right], & i \neq j \\ 0, & i = j \end{cases} \quad [41]$$

Therefore, the condition for evolutionary success of cooperation is given by letting $\left.\frac{d}{d\delta}\right|_{\delta=0}\rho_C > 0$, where

$$\begin{aligned}\left.\frac{d}{d\delta}\right|_{\delta=0}\rho_C = -\frac{c}{N^2}\sum_{t=0}^{\infty}\sum_{i,j=1}^{N}\pi_i p_{ij}^{\mathrm{IM}(2)} q_j(t)\,\mathbb{P}_{(i,j)}\left[T^{\mathrm{coal}} > t\right] \\ + \frac{b}{N^2}\left(-\sum_{t=0}^{\infty}\sum_{i,j=1}^{N}\pi_i q_{ij}(t)\,\mathbb{P}_{(i,j)}\left[T^{\mathrm{coal}} > t\right] + \sum_{t=0}^{\infty}\sum_{i,j,k=1}^{N}\pi_i p_{ij}^{\mathrm{IM}(2)} q_{jk}(t)\,\mathbb{P}_{(i,k)}\left[T^{\mathrm{coal}} > t\right]\right),\end{aligned} \quad [42]$$

and the critical benefit-to-cost ratio $(b/c)^*$ is obtained correspondingly.

B. Pairwise-comparison (PC). Under PC updating, an individual is chosen uniformly-at-random to update its strategy. It then compares its own payoff with that of a single, randomly chosen neighbor. The focal individual adopts the neighbor's strategy or retains its current strategy with the probability proportional to the corresponding fitness. In this case, the probability of an individual i transmitting its strategy to another individual j $(i \neq j)$ is

$$e_{ij}(\mathbf{x}, t) = \frac{1}{N} p_{ji}\frac{F_i(\mathbf{x}, t)}{F_i(\mathbf{x}, t) + F_j(\mathbf{x}, t)}, \quad [43]$$

otherwise it retains its own strategy with probability

$$e_{ii}(\mathbf{x}, t) = \frac{1}{N}\sum_{j=1}^{N} p_{ij}\frac{F_i(\mathbf{x}, t)}{F_i(\mathbf{x}, t) + F_j(\mathbf{x}, t)}, \quad [44]$$

Accordingly, we have

$$\begin{aligned}\left.\frac{d}{d\delta}\right|_{\delta=0}\rho_C = \frac{c}{2N}\Bigg(&\sum_{t=0}^{\infty}\sum_{i=1}^{N}\pi_i q_i\left(t\right)\left(\frac{1}{N}-\mathbb{E}^{\circ}\left[x_i\left(t\right)\mid \mathbf{x}\left(0\right)\sim\mu_C\right]\right)\\ &-\sum_{t=0}^{\infty}\sum_{i,j=1}^{N}\pi_i p_{ij} q_j\left(t\right)\left(\frac{1}{N}-\mathbb{E}^{\circ}\left[x_i\left(t\right)x_j\left(t\right)\mid \mathbf{x}\left(0\right)\sim\mu_C\right]\right)\Bigg)\\ +\frac{b}{2N}\Bigg(&-\sum_{t=0}^{\infty}\sum_{i,j=1}^{N}\pi_i q_{ij}\left(t\right)\left(\frac{1}{N}-\mathbb{E}^{\circ}\left[x_i\left(t\right)x_j\left(t\right)\mid \mathbf{x}\left(0\right)\sim\mu_C\right]\right)\\ &+\sum_{t=0}^{\infty}\sum_{i,j,k=1}^{N}\pi_i p_{ij} q_{jk}\left(t\right)\left(\frac{1}{N}-\mathbb{E}^{\circ}\left[x_i\left(t\right)x_k\left(t\right)\mid \mathbf{x}\left(0\right)\sim\mu_C\right]\right)\Bigg).\end{aligned} \quad [45]$$

For PC updating, the recurrence relationship of the coalescence probability is given by

$$\mathbb{P}_{(i,j)}\left[T^{\text{coal}}>t\right]=\begin{cases}\frac{1}{2N}\sum_{k=1}^{N}p_{ik}\mathbb{P}_{(k,j)}\left[T^{\text{coal}}>t-1\right]+\frac{1}{2N}\sum_{k=1}^{N}p_{jk}\mathbb{P}_{(k,i)}\left[T^{\text{coal}}>t-1\right] & \\ +\frac{N-1}{N}\mathbb{P}_{(i,j)}\left[T^{\text{coal}}>t-1\right], & i\neq j\\ 0, & i=j\end{cases} \quad [46]$$

Therefore, we have

$$\begin{aligned}\left.\frac{d}{d\delta}\right|_{\delta=0}\rho_C =& -\frac{c}{N^2}\sum_{t=0}^{\infty}\sum_{i,j=1}^{N}\pi_i p_{ij} q_j\left(t\right)\mathbb{P}_{(i,j)}\left[T^{\text{coal}}>t\right]\\ &+\frac{b}{N^2}\left(-\sum_{t=0}^{\infty}\sum_{i,j=1}^{N}\pi_i q_{ij}\left(t\right)\mathbb{P}_{(i,j)}\left[T^{\text{coal}}>t\right]+\sum_{t=0}^{\infty}\sum_{i,j,k=1}^{N}\pi_i p_{ij} q_{jk}\left(t\right)\mathbb{P}_{(i,k)}\left[T^{\text{coal}}>t\right]\right),\end{aligned} \quad [47]$$

and the critical benefit-to-cost ratio is obtained by letting $\left.\frac{d}{d\delta}\right|_{\delta=0}\rho_C>0$.

C. Birth-death (BD) process. For BD updating, an individual is chosen to give a birth with a probability proportional to its own fitness, an then randomly replace one of its neighbors with its offspring. Therefore, the probability for individual i transmits its strategy to j is

$$e_{ij}\left(\mathbf{x},t\right)=p_{ij}\frac{F_i\left(\mathbf{x},t\right)}{\sum_{k=1}^{N}F_k\left(\mathbf{x},t\right)}, \quad [48]$$

Accordingly, we have

$$\begin{aligned}\left.\frac{d}{d\delta}\right|_{\delta=0}\rho_C = \frac{c}{N}\Bigg(&\sum_{t=0}^{\infty}\sum_{i=1}^{N}\pi_i p_{ji} q_j\left(t\right)\left(\frac{1}{N}-\mathbb{E}^{\circ}\left[x_i\left(t\right)\mid \mathbf{x}\left(0\right)\sim\mu_C\right]\right)\\ &-\sum_{t=0}^{\infty}\sum_{i,j=1}^{N}\pi_i p_{ji} q_j\left(t\right)\left(\frac{1}{N}-\mathbb{E}^{\circ}\left[x_i\left(t\right)x_j\left(t\right)\mid \mathbf{x}\left(0\right)\sim\mu_C\right]\right)\Bigg)\\ +\frac{b}{N}\Bigg(&\sum_{t=0}^{\infty}\sum_{i,j=1}^{N}\pi_i p_{ji} q_{jk}\left(t\right)\left(\frac{1}{N}-\mathbb{E}^{\circ}\left[x_i\left(t\right)x_k\left(t\right)\mid \mathbf{x}\left(0\right)\sim\mu_C\right]\right)\\ &-\sum_{t=0}^{\infty}\sum_{i,j,k=1}^{N}\pi_i p_{ji} q_{ik}\left(t\right)\left(\frac{1}{N}-\mathbb{E}^{\circ}\left[x_i\left(t\right)x_k\left(t\right)\mid \mathbf{x}\left(0\right)\sim\mu_C\right]\right)\Bigg).\end{aligned} \quad [49]$$

For BD updating, the recurrence relationship of the coalescence probability is given by

$$\mathbb{P}_{(i,j)}\left[T^{\text{coal}}>t\right]=\begin{cases}\frac{1}{N}\sum_{k=1}^{N}p_{ki}\mathbb{P}_{(k,j)}\left[T^{\text{coal}}>t-1\right]+\frac{1}{N}\sum_{k=1}^{N}p_{kj}\mathbb{P}_{(k,i)}\left[T^{\text{coal}}>t-1\right] & \\ +\left(1-\frac{1}{N}\sum_{k=1}^{N}p_{ki}-\frac{1}{N}\sum_{k=1}^{N}p_{kj}\right)\mathbb{P}_{(i,j)}\left[T^{\text{coal}}>t-1\right], & i\neq j\\ 0, & i=j\end{cases} \quad [50]$$

Therefore, we have

$$\left.\frac{d}{d\delta}\right|_{\delta=0}\rho_C=-\frac{c}{N^2}\sum_{t=0}^{\infty}\sum_{i,j=1}^{N}\pi_i p_{ji} q_j\left(t\right)\mathbb{P}_{(i,j)}\left[T^{\text{coal}}>t\right]+\frac{b}{N^2}\sum_{t=0}^{\infty}\sum_{i,j,k=1}^{N}\pi_i p_{ji}\left(q_{jk}\left(t\right)-q_{ik}\left(t\right)\right)\mathbb{P}_{(i,k)}\left[T^{\text{coal}}>t\right], \quad [51]$$

and the critical benefit-to-cost ratio is obtained by letting $\left.\frac{d}{d\delta}\right|_{\delta=0}\rho_C>0$.

S5. Temporal hypernetworks

In this section, we study the evolution of cooperation on temporal higher-order networks. Here we apply hypernetworks to capture the higher-order interactions among individuals at each time step, where the group interactions are captured by the hyperedges linking multiple individuals. We use $I_{i\alpha}(t)$ to indicate that the individual i play a public goods game on the hyperedge α. Analogously to the definition of the single-step random walk from i to j on the snapshot at time t ($q_{ij}(t)$) in the scenarios of traditional pairwise interactions (Supporting Information Text S1), we define $q_{i\alpha}(t) = I_{i\alpha}(t)/n_i(t)$ when $n_i(t) > 0$ as the probability of a single step random walk from individual i to an hyperedge α, where $n_i(t) = \sum_\alpha I_{i\alpha}(t)$ is the number of hyperedges that individual i is involved at time t. Otherwise when $n_i(t) = 0$ we have $q_{i\alpha}(t) = 0$. Subsequently, we can define the random walk from hyperedge α to individual j with $\check{q}_{\alpha j}(t) = I_{j\alpha}(t)/I_\alpha(t)$, where $I_\alpha(t) = \sum_{i=1}^N I_{i\alpha}(t)$ indicates the size of the hyperedge α at this time (the number of individuals in this group). Note that we use $\check{q}_{\alpha i}$ for random walks from hyperedges to nodes to distinguish from the case of moving from nodes to hyperedges ($q_{i\alpha}$).

We explore the evolutionary dynamics with public goods games over time, where the games occur on hyperedges at each snapshot. In each round of games, individuals participate into the public goods games on hyperedges they involved and accumulate the corresponding payoffs. The payoff for defectors and cooperators on an hyperedge α with group size I_α is given by $u_D = Rcn_C/I_\alpha$ and $u_C = Rcn_C/I_\alpha - c$, where n_C represents the number of cooperators in the group, and R is the synergy factor of the public goods game. Therefore, the average payoff of individual i (the payoff the individual accumulate from each group, divided by the total number of hyperedges it involved) by

$$u_i(\mathbf{x}, t) = -cq_i(t)x_i + cR \sum_{\alpha \in E(t)} q_{i\alpha}(t) \frac{1}{I_\alpha(t)} \sum_{k=1}^N I_{k\alpha}(t) x_k, \qquad [52]$$

where $q_i(t) = \sum_{\alpha \in E(t)} q_{i\alpha}(t)$, here $E(t)$ represents the set of hyperedges at time step t.

After each round of public goods games, an individual is uniformly at random selected from the population to update its strategy following the death-birth process. It will copy the strategy of its neighbors with probability proportional to the corresponding fitness. Note that the static replacement network here captures the social relationship of the populations, where there is an edge between two nodes if they have participated at least once in a common public goods game, namely they are involved at least once in a common hyperedge over time. In other words, the aggregated social relationship captures who can imitate strategies from whom, which can be obtained by aggregating all hypernetworks over snapshots and connecting each pair of nodes within each hyperedge (Fig. 4D in the main text).

Following the procedure of the calculation of fixation probability shown in Supporting Information Text S1-S3, we can obtain

$$\begin{aligned} \left.\frac{d}{d\delta}\right|_{\delta=0} \rho_C = & -\frac{c}{N^2} \sum_{t=0}^{\infty} \sum_{i,j=1}^{N} \pi_i p_{ij}^{(2)} q_j(t) \mathbb{P}_{(i,j)} \left[T^{\text{coal}} > t\right] \\ & + \frac{cR}{N^2} \sum_{t=0}^{\infty} \sum_{i,j,k=1}^{N} \pi_i p_{ij}^{(2)} \sum_{\alpha \in E(t)} q_{j\alpha}(t) \check{q}_{\alpha k}(t) \mathbb{P}_{(i,k)} \left[T^{\text{coal}} > t\right] \\ & - \frac{cR}{N^2} \sum_{t=0}^{\infty} \sum_{i,j=1}^{N} \pi_i \sum_{\alpha \in E(t)} q_{i\alpha}(t) \check{q}_{\alpha j}(t) \mathbb{P}_{(i,j)} \left[T^{\text{coal}} > t\right], \end{aligned} \qquad [53]$$

By letting $\left.\frac{d}{d\delta}\right|_{\delta=0} \rho_C > 0$ in Eq. 53, we can obtain the critical synergy factor R^*, above which cooperation is favored by selection on temporal hypernetworks, which is given by

$$R^* = \frac{\sum_{t=0}^{\infty} \sum_{i,j=1}^{N} \pi_i p_{ij}^{(2)} q_j(t) \mathbb{P}_{(i,j)} \left[T^{\text{coal}} > t\right]}{\sum_{t=0}^{\infty} \sum_{i,j,k=1}^{N} \pi_i p_{ij}^{(2)} \tilde{q}_{jk}(t) \mathbb{P}_{(i,k)} \left[T^{\text{coal}} > t\right] - \sum_{t=0}^{\infty} \sum_{i,j=1}^{N} \pi_i \tilde{q}_{ij}(t) \mathbb{P}_{(i,j)} \left[T^{\text{coal}} > t\right]}, \qquad [54]$$

where $\tilde{q}_{ij}(t) = \sum_{\alpha \in E(t)} q_{i\alpha}(t) \check{q}_{\alpha j}(t)$. This result shares the similar form of evolutionary dynamics on traditional temporal pairwise interactions in Eq. 23, with the only difference of replacing the $q_{ij}(t)$ by $\tilde{q}_{ij}(t)$ on temporal hypernetworks.

Subsequently, by applying the mean-field approximation following the approach shown in Supporting Information Text S3, we obtain the approximated result for the critical synergy factor

$$R^* \approx \frac{Q + \varepsilon_c}{\tilde{Q} + L - 2\overline{q} + \varepsilon_b}, \qquad [55]$$

where $\tilde{Q} = \sum_{m=1}^{\infty} \sum_{T=0}^{m} \sum_{i=1}^{N} \pi_i \tilde{q}_{ii}(\lfloor TN/2 \rfloor) \mathbb{P}[\tau = m]$, and other terms has the same form as those in Eq. 3 in the main text by replacing q_{ij} by $\tilde{q}_{ij}$.

In the traditional pairwise interactions, there are no self-loops on each snapshot, thus the term $\tilde{Q}$ is omitted in Eq. 3 in the main text. While for public goods games on hyperedges, there exists the path from an individual going through a hyperedge to itself, because cooperators will also receive a return from the common pool. Since $\tilde{Q}$ is of greater magnitude than L, which undergoes a further step random walk on the replacement network, the dominant factor of R^* becomes Q and $\tilde{Q}$. Correspondingly, we can define the priority of hubs by $\tilde{H} := q/\sum_{i=1}^N \pi_i \tilde{q}_{ii}$ for temporal hypernetworks instead of q/l for pairwise interactions in the main text. By implementing the priority of hubs $\tilde{H}$ in ascending ordering on real datasets, the rearranged sequence of temporal hypernetworks can actually promote the emergence of cooperation with lower R^* (Fig. 4F-H in the main text).

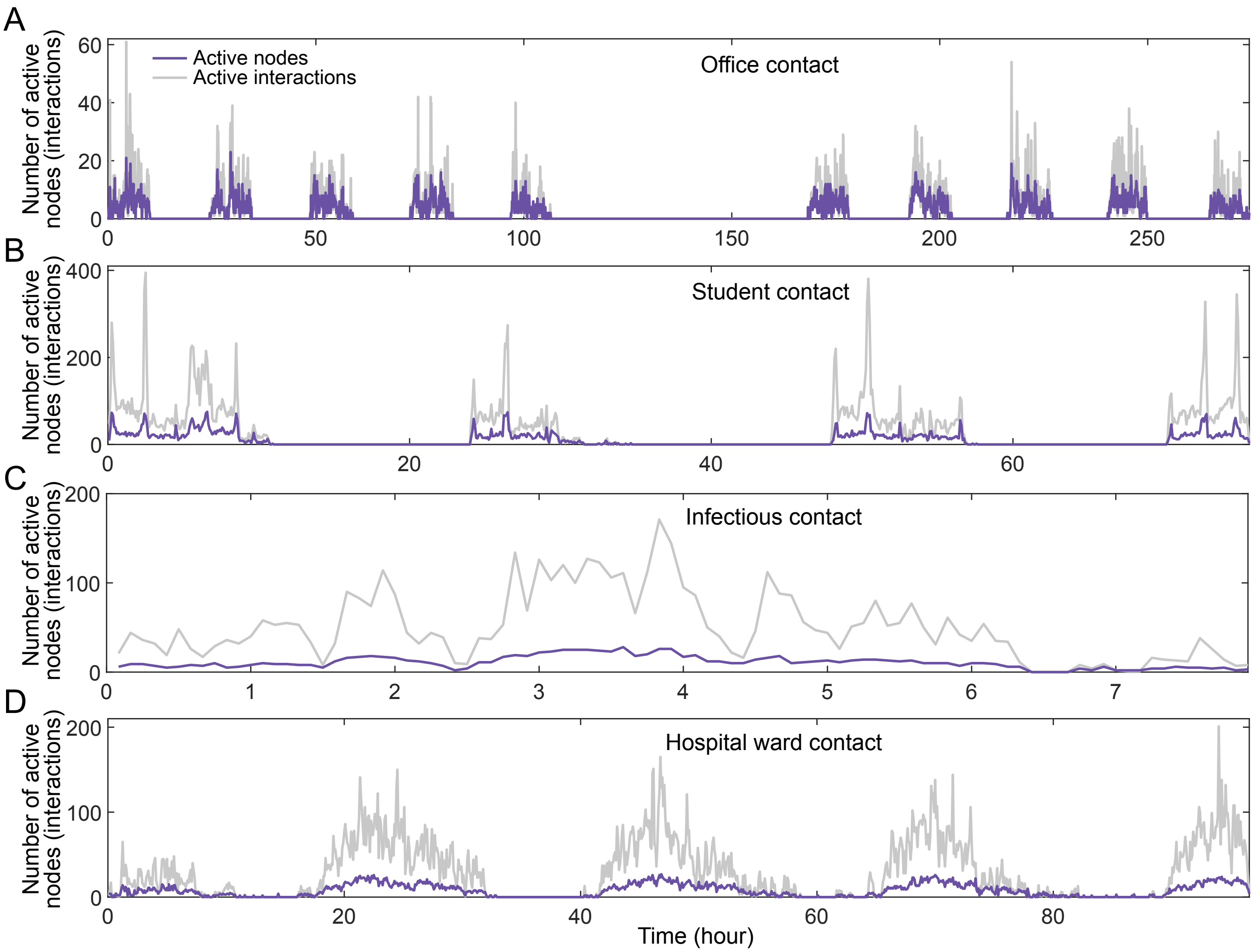

Fig. S1. Illustration of the number of active nodes and interactions over time on empirical datasets. We show that the number of active nodes (purple line) and interactions (gray line) over time of empirical datasets collected from contacts in office (12) (*A*), high school (13) (*B*), exhibition (14) (*C*), and hospital (15) (*D*), respectively. Here the time window $\Delta t = 5$ minutes.

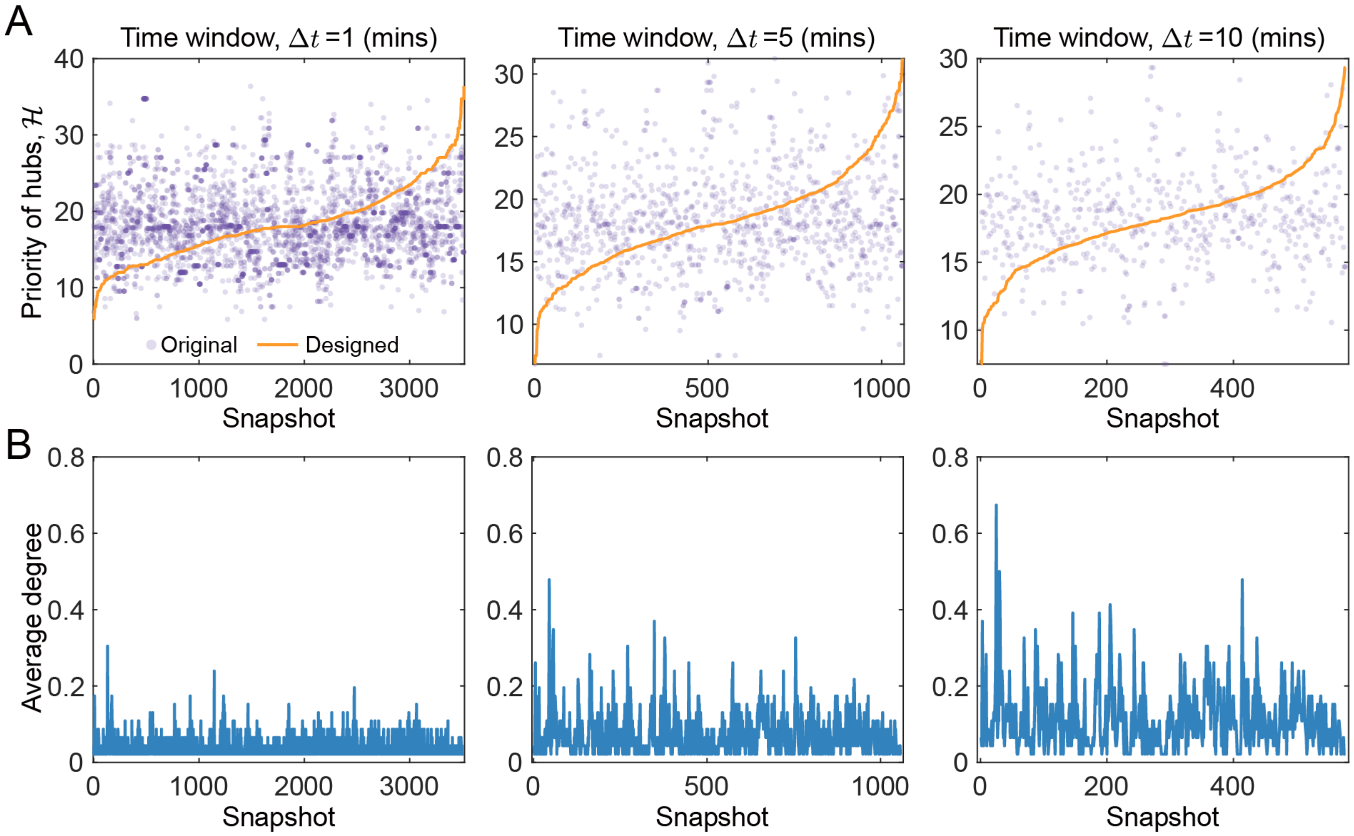

Fig. S2. Designing temporal interactions on empirical networks collected from office contact. (*A*) We show the priority of hubs $\mathcal{H}$ over snapshots with the original (purple dot) and designed (golden line) ordering in empirical networks collected from contacts in an office (12). The corresponding temporal networks are constructed by aggregating interactions with time window of $\Delta t = 1, 5, 10$ minutes and removing the snapshots with no interactions. The original empirical networks exhibit temporal randomness in the priority of hubs over the snapshots, which are rearranged in ascending ordering in the designed sequences. The average degree of each snapshot in empirical temporal networks is shown in (*B*).

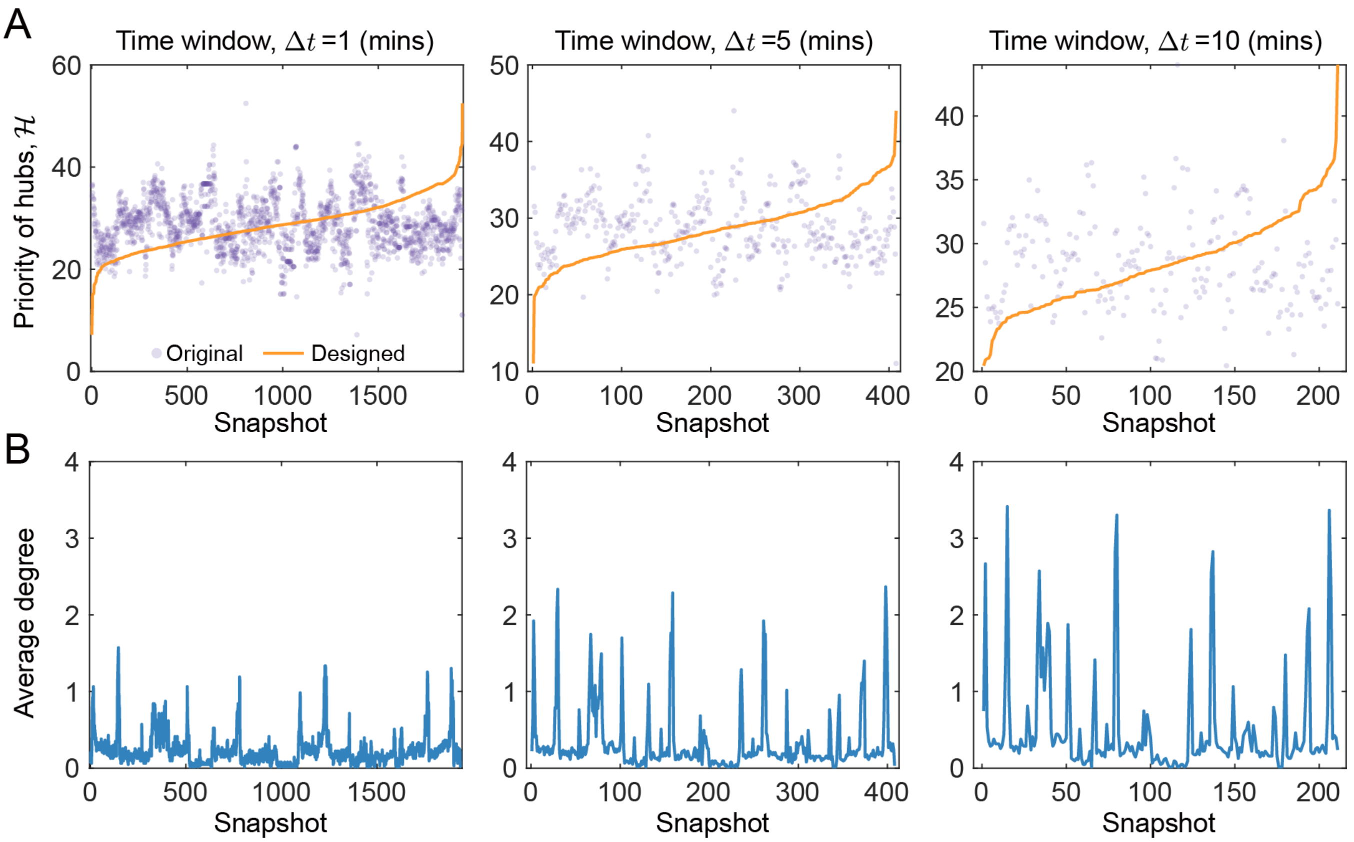

Fig. S3. Designing temporal interactions on empirical networks collected from student contact. (*A*) We show the priority of hubs $\mathcal{H}$ over snapshots with the original (purple dot) and designed (golden line) ordering in empirical networks collected from contacts in a high school (13). The corresponding temporal networks are constructed by aggregating interactions with time window of $\Delta t = 1, 5, 10$ minutes and removing the snapshots with no interactions. The original empirical networks exhibit temporal randomness in the priority of hubs over the snapshots, which are rearranged in ascending ordering in the designed sequences. The average degree of each snapshot in empirical temporal networks is shown in (*B*).

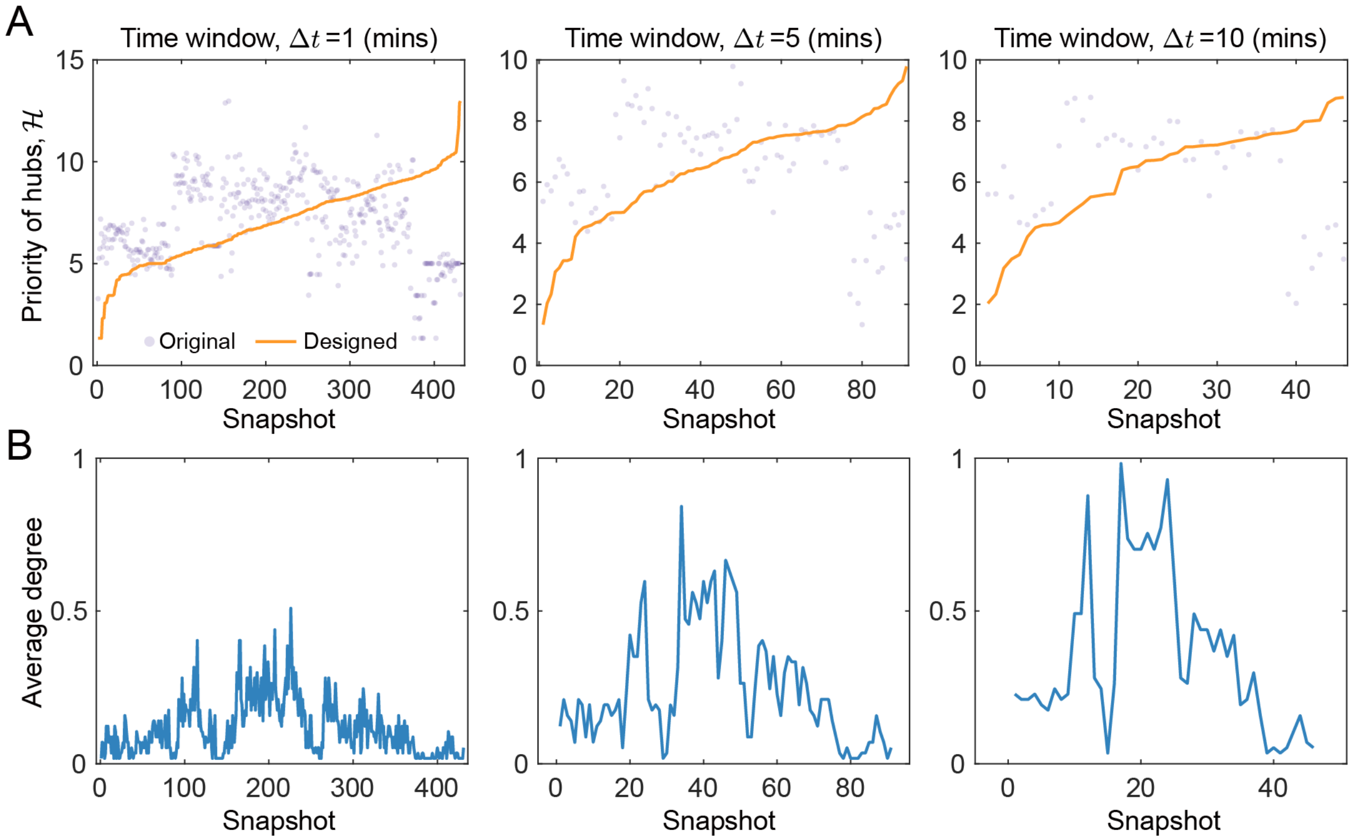

Fig. S4. Designing temporal interactions on empirical networks collected from infectious contact. (*A*) We show the priority of hubs $\mathcal{H}$ over snapshots with the original (purple dot) and designed (golden line) ordering in empirical networks collected from contacts in an artscience exhibition (14). The corresponding temporal networks are constructed by aggregating interactions with time window of $\Delta t = 1, 5, 10$ minutes and removing the snapshots with no interactions. The original empirical networks exhibit temporal randomness in the priority of hubs over the snapshots, which are rearranged in ascending ordering in the designed sequences. The average degree of each snapshot in empirical temporal networks is shown in (*B*).

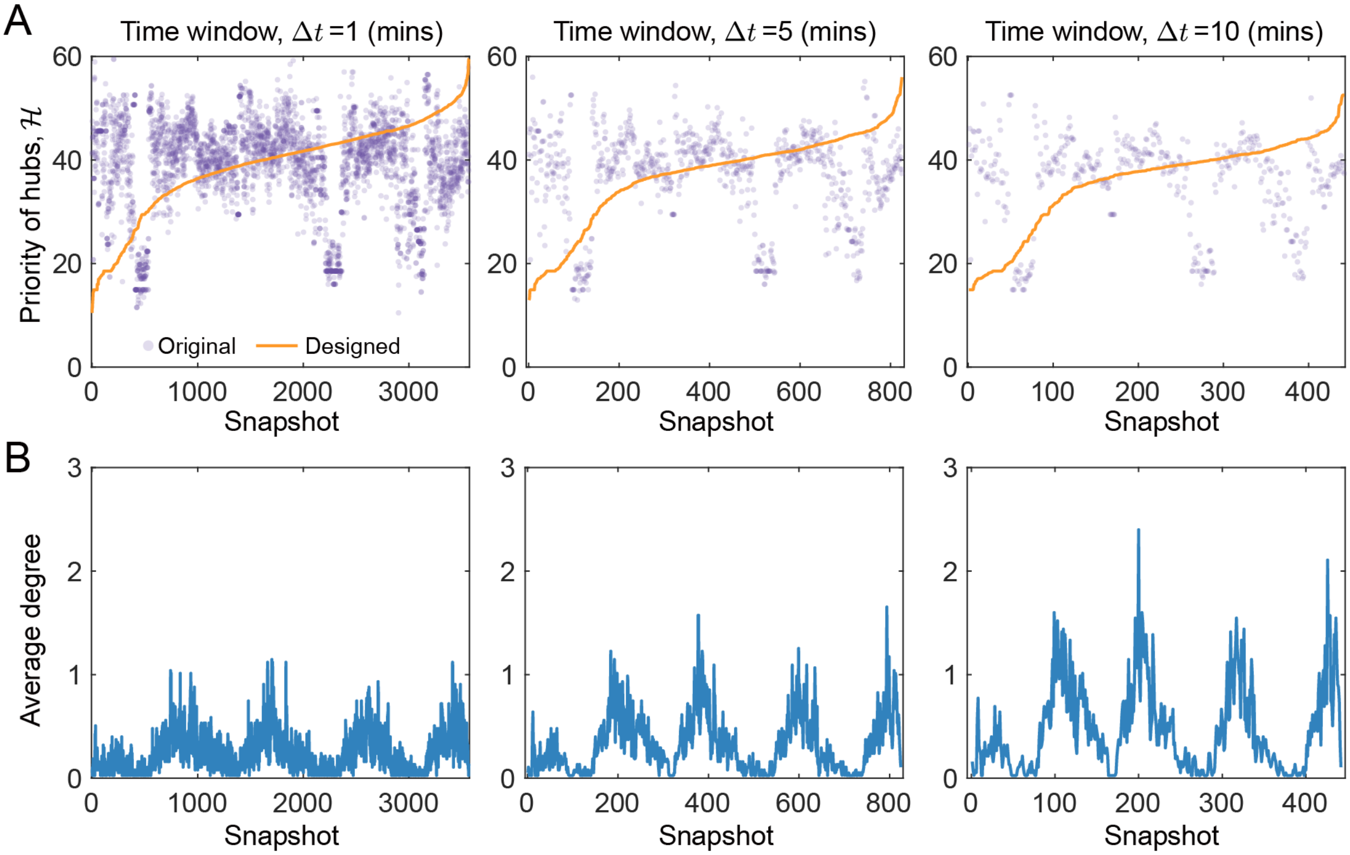

Fig. S5. Designing temporal interactions on empirical networks collected from hospital ward contact. (*A*) We show the priority of hubs $\mathcal{H}$ over snapshots with the original (purple dot) and designed (golden line) ordering in empirical networks collected from contacts in a hospital (15). The corresponding temporal networks are constructed by aggregating interactions with time window of $\Delta t = 1, 5, 10$ minutes and removing the snapshots with no interactions. The original empirical networks exhibit temporal randomness in the priority of hubs over the snapshots, which are rearranged in ascending ordering in the designed sequences. The average degree of each snapshot in empirical temporal networks is shown in (*B*).

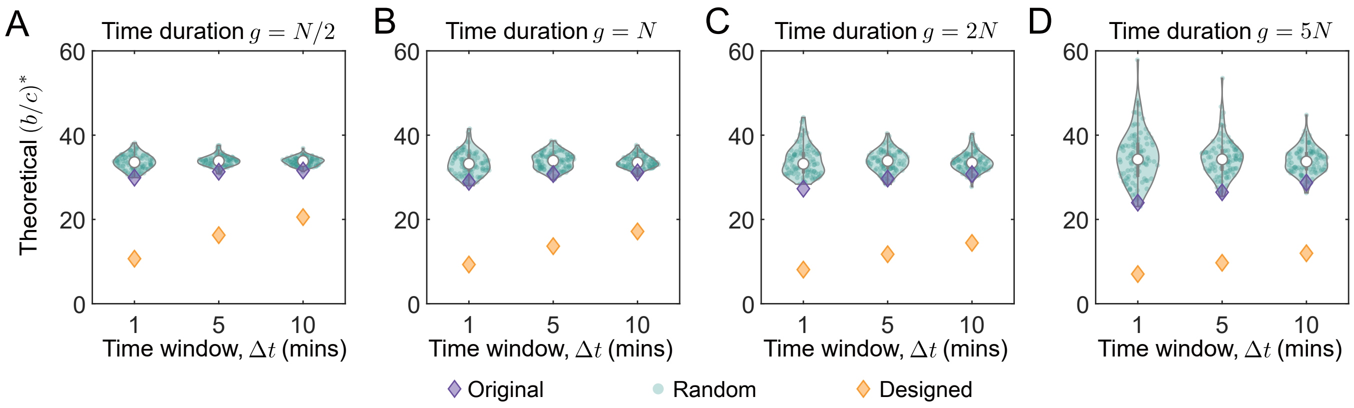

Fig. S6. Illustration of critical ratio $(b/c)^*$ with different time duration on temporal interactions collected from office contact. In the main text, each snapshot lasts for N steps of games and strategy updates, where N is the population size so that each individual updates its strategy once on average over the snapshot. Here we consider different time duration $g = N/2, N, 2N, 5N$ on each snapshot, and calculate the critical ratio $(b/c)^*$ to confirm the effectiveness of our designed temporal interactions collected from office contact (12) in (*A*)-(*D*), respectively. The $(b/c)^*$ with natural (designed) ordering is marked in purple (golden) diamond. As a control group, we plot the distribution of the random permuted snapshot sequence over 100 samples in cyan dots and shadows in different settings of time duration g.

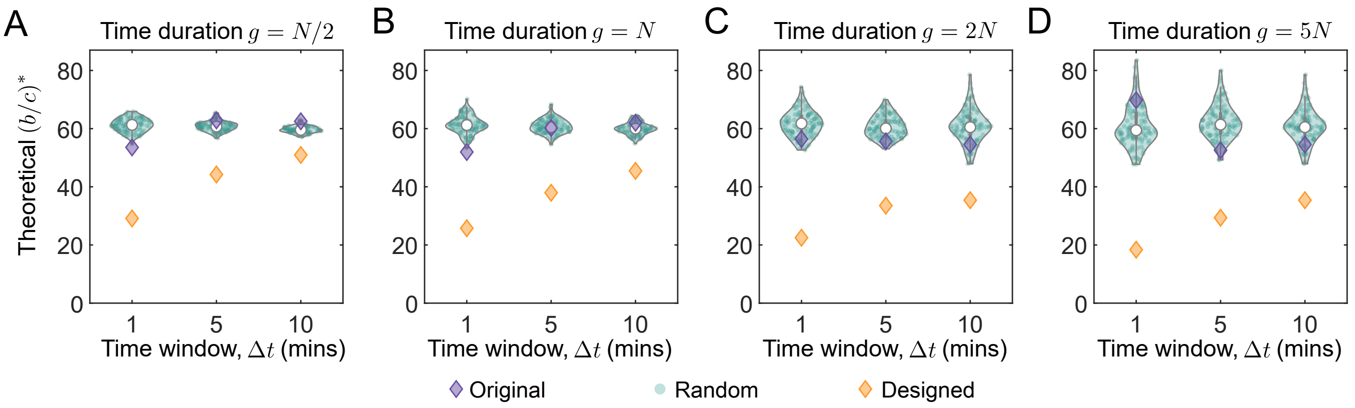

Fig. S7. Illustration of critical ratio $(b/c)^*$ with different time duration on temporal interactions collected from student contact. In the main text, each snapshot lasts for N steps of games and strategy updates, where N is the population size so that each individual updates its strategy once on average over the snapshot. Here we consider different time duration $g = N/2, N, 2N, 5N$ on each snapshot, and calculate the critical ratio $(b/c)^*$ to confirm the effectiveness of our designed temporal interactions collected from student contact (13) in (*A*)-(*D*), respectively. The $(b/c)^*$ with natural (designed) ordering is marked in purple (golden) diamond. As a control group, we plot the distribution of the random permuted snapshot sequence over 100 samples in cyan dots and shadows in different settings of time duration g.

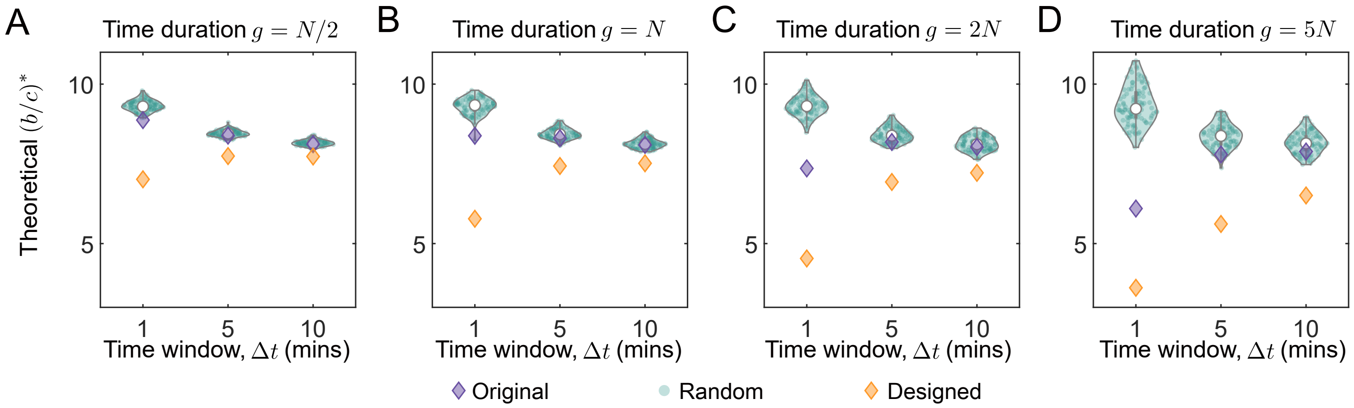

Fig. S8. Illustration of critical ratio $(b/c)^*$ with different time duration on temporal interactions collected from infectious contact. In the main text, each snapshot lasts for N steps of games and strategy updates, where N is the population size so that each individual updates its strategy once on average over the snapshot. Here we consider different time duration $g = N/2, N, 2N, 5N$ on each snapshot, and calculate the critical ratio $(b/c)^*$ to confirm the effectiveness of our designed temporal interactions collected from infectious contact (14) in (*A*)-(*D*), respectively. The $(b/c)^*$ with natural (designed) ordering is marked in purple (golden) diamond. As a control group, we plot the distribution of the random permuted snapshot sequence over 100 samples in cyan dots and shadows in different settings of time duration g.

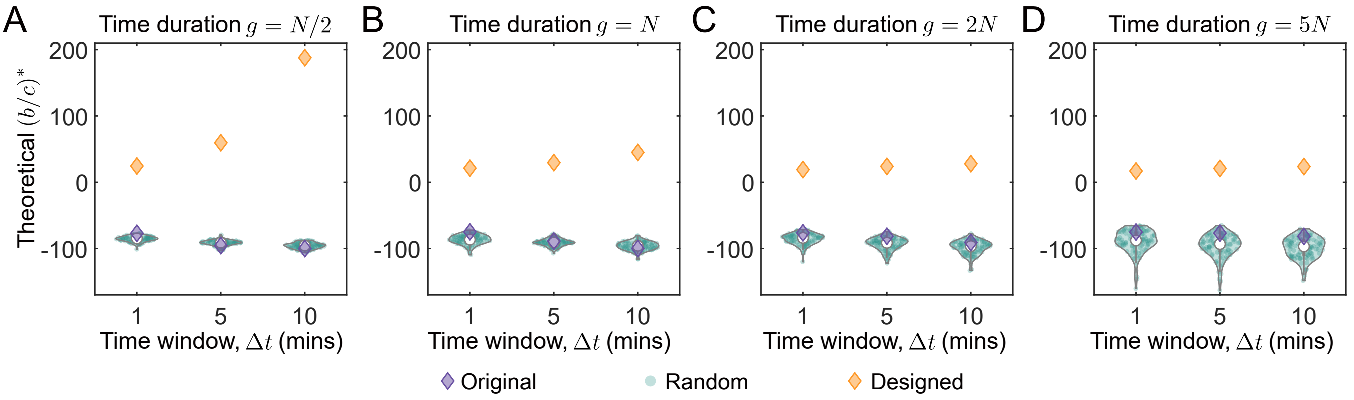

Fig. S9. Illustration of critical ratio $(b/c)^*$ with different time duration on temporal interactions collected from hospital ward contact. In the main text, each snapshot lasts for N steps of games and strategy updates, where N is the population size so that each individual updates its strategy once on average over the snapshot. Here we consider different time duration $g = N/2, N, 2N, 5N$ on each snapshot, and calculate the critical ratio $(b/c)^*$ to confirm the effectiveness of our designed temporal interactions collected from hospital ward contact (15) in (*A*)-(*D*), respectively. The $(b/c)^*$ with natural (designed) ordering is marked in purple (golden) diamond. As a control group, we plot the distribution of the random permuted snapshot sequence over 100 samples in cyan dots and shadows in different settings of time duration g.

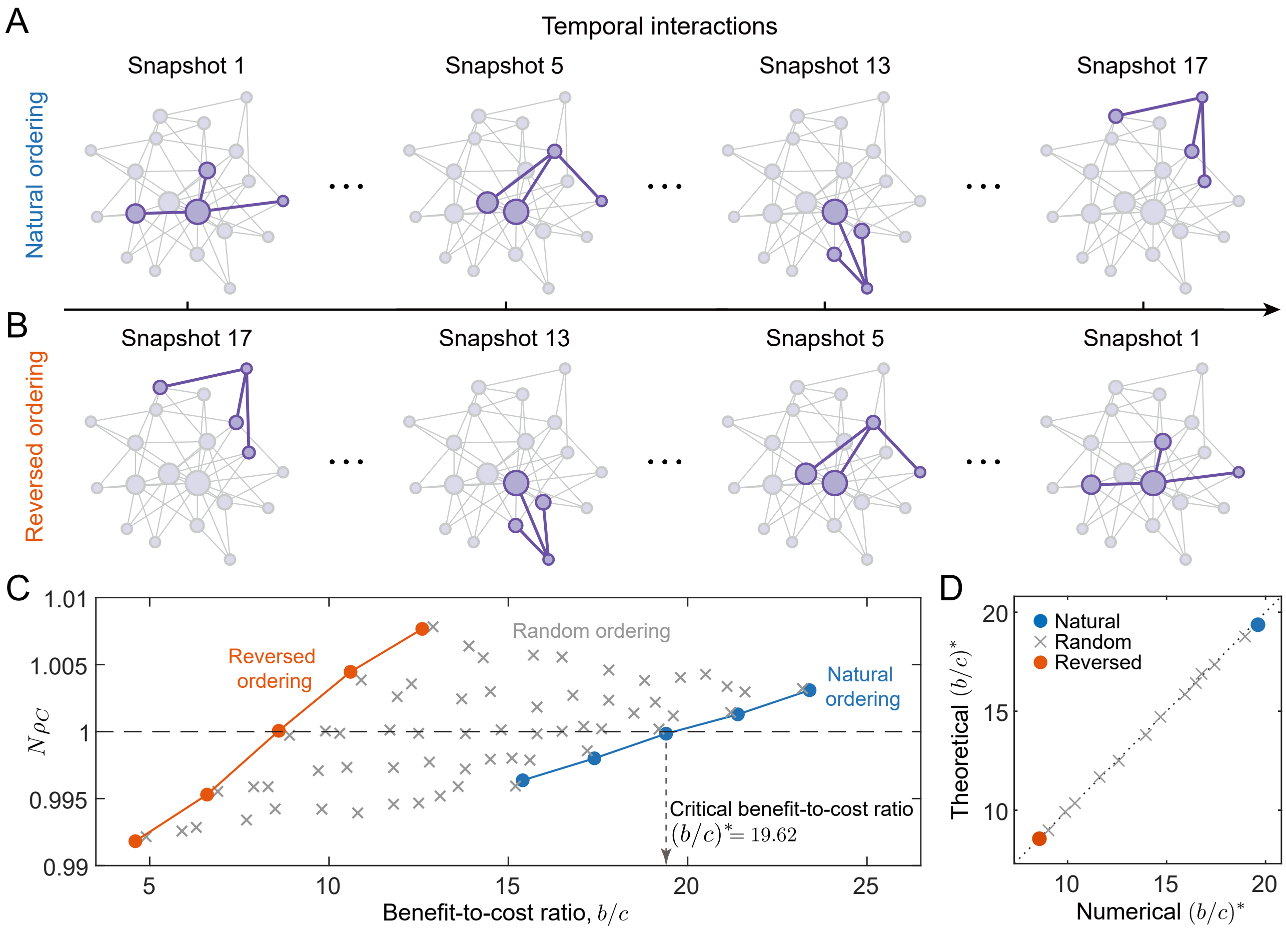

Fig. S10. Effect of interaction ordering on the emergence of cooperation. (*A*) We generate temporal interactions under Barabási-Albert model (16), where a new node is added to the network and connected to 3 different existing nodes at each step. We take the newly added edges and nodes of both ends as the interaction network (purple) in each snapshot, which lasts for $N = 20$ time steps. We illustrate the interactions in snapshots 1, 5, 13, and 17. The replacement network (gray) is the static Barabási-Albert network with N nodes. The active nodes (purple) play games pairwise on the active links (purple), and the replacement of strategies occurs on the underlying aggregated network. Each snapshot lasts for N time steps (equal to the population size), ensuring that each individual updates its strategy once, on average, over the snapshot. (*B*) We show the interactions with reversed time-ordering. (*C*) We show the fixation probability of cooperation (ρ_C) as a function of the benefit-to-cost ratio (b/c) for natural (blue circle), random (gray cross), and reversed (orange circle) ordering. The critical benefit-to-cost ratio, $(b/c)^*$, occurs when the corresponding curve of ρ_C intersects the horizontal line ($\rho_C = 1/N$), indicating neutral drift. $(b/c)^*$ for the natural ordering is highlighted in blue, which is slightly less than 20. (*D*) We show that the simulation results are in good agreement with theoretical predictions calculated from Eq. 1 in the main text.

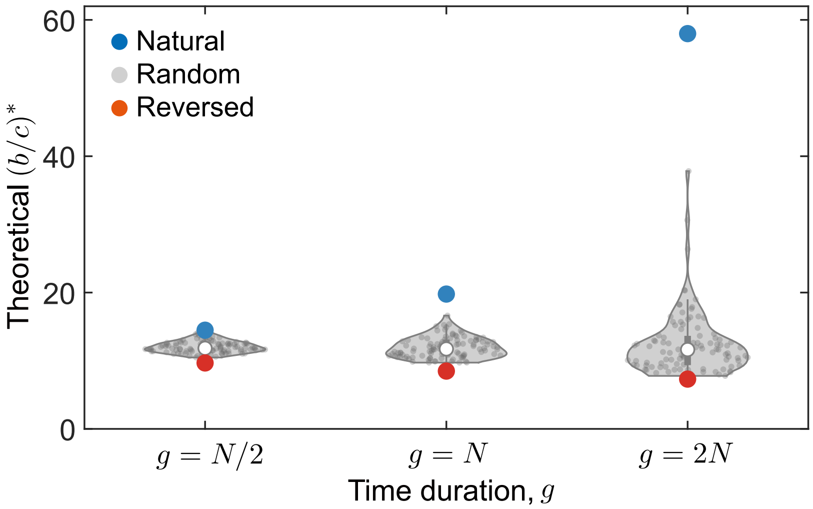

Fig. S11. Effect of interaction ordering on the emergence of cooperation with different time durations. In the main text, each snapshot lasts for N steps of games and strategy updates, where N is the population size so that each individual updates its strategy once on average over the snapshot. Here we consider different time duration $g = N/2, N, 2N$ on each snapshot, and calculate the critical ratio $(b/c)^*$ on temporal interactions generated from Barabási-Albert (BA) (16). The $(b/c)^*$ with natural (reversed) ordering is marked in blue (red) dot. As a control group, we plot the distribution of the random permuted snapshot sequence over 100 samples in gray dots and shadows in different settings of time duration g, with median values shown in white dots. Other parameters are the same as those in Fig. 2 in the main text.

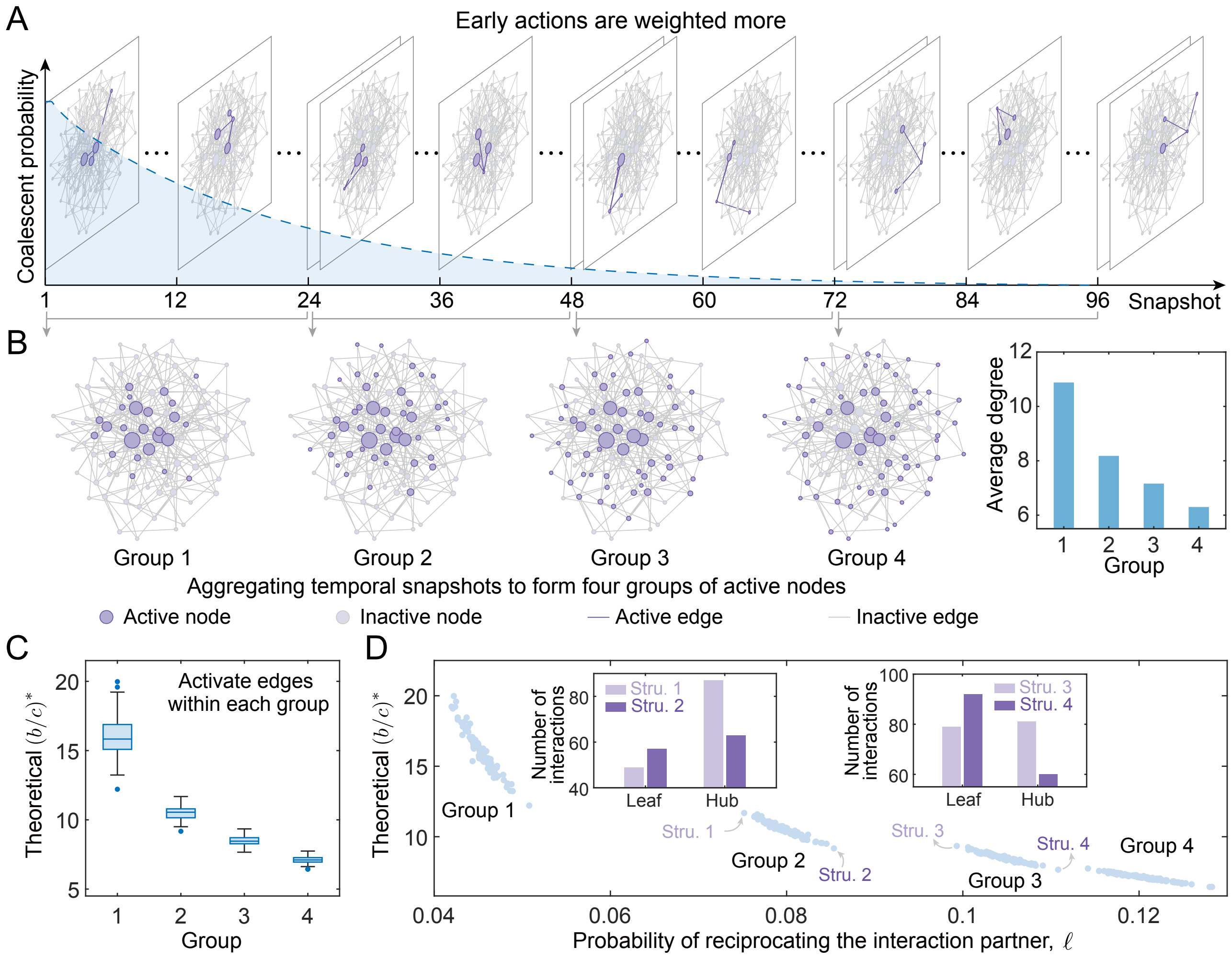

Fig. S12. A simple rule for promoting cooperation in temporal and static networks. (*A*) We show a sequence of snapshots in temporal interactions generated under preferential attachment (16) with 100 nodes which governs that early active nodes (purple) tend to have large degrees. The coalescent probability (dashed line) governing the dynamics of strategy dispersal decays exponentially, indicating that the earlier interactions are weighted more in the evolution (Eq. 3 in the main text). (*B*) We divide the temporal snapshots into four groups of active nodes, where the average degree for each group decreases accordingly (*C*). (*D*) The interaction edges (purple) in each group are selected with probability $p = 0.5$ from the underlying replacement networks, and the group with more hubs yields higher $(b/c)^*$. (*E*) We show $(b/c)^*$ as a function of the probability of reciprocating the interaction partner (ℓ). For Group 2 and Group 3, we divide the nodes into hubs (with top 17% degree) and leaves, and show the number of interactions accordingly on structures with the highest and lowest $(b/c)^*$ respectively, where the fewer interactions the hubs involved, the lower the value of $(b/c)^*$ is.

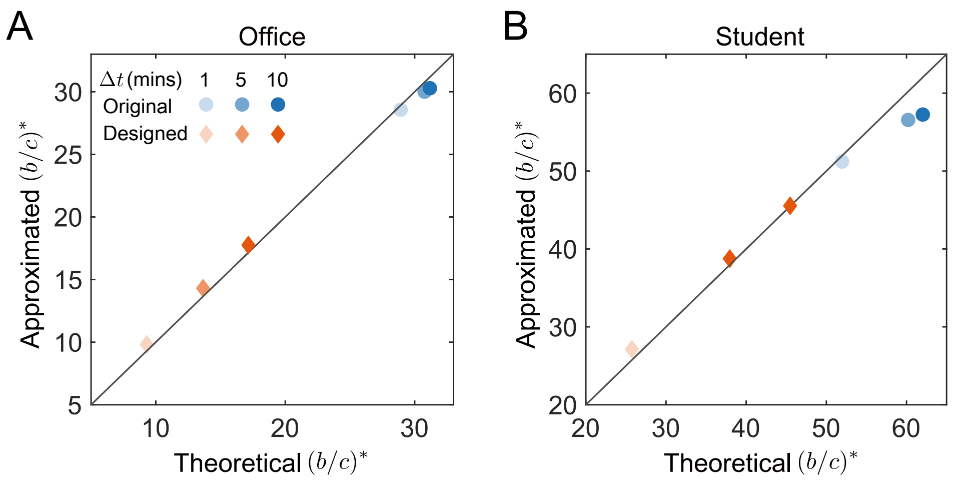

Fig. S13. Illustration of the accuracy of mean-field approximation on empirical temporal networks. We calculate the critical ratio, $(b/c)^*$, using a mean-field approximation on temporal interactions with natural (blue dots) and designed (orange diamond) ordering for two empirical datasets collected from contacts in *A*, an office (12) and *B*, a high school (13). A remarkable accuracy is presented among all different settings of time windows ($\Delta t = 1, 5, 10$ (minutes)).

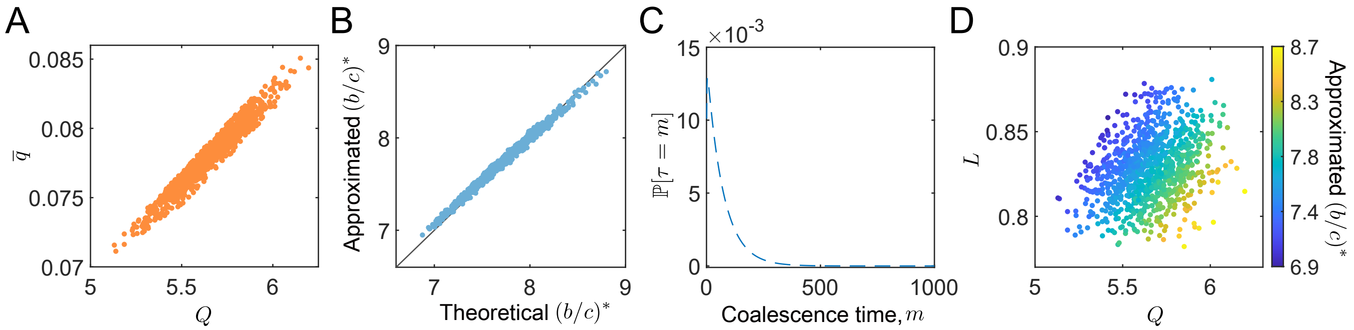

Fig. S14. Intuition for designing temporal interactions using a mean-field approximation. (*A*) Here we show that Q and $\overline{q}$ are positively related, which can also be obtained from Eq. 3. (*B*) We confirm the accuracy of the approximation of the critical ratio $(b/c)^*$ in Eq. 3 in the main text on a temporal network generated by preferential attachment. We observe remarkable accuracy of the mean-field approximation approach with 1000 realizations of randomizing the ordering of the temporal interactions. (*C*) We further present the exponential decay of the average coalescent probability ($\mathbb{P}[\tau = m]$) as a function of m. (*D*) A small Q and a large L result in a lower critical ratio, which facilitates the emergence of cooperation. The temporal network contains 100 nodes and has a degree of 6 on the underlying replacement network.

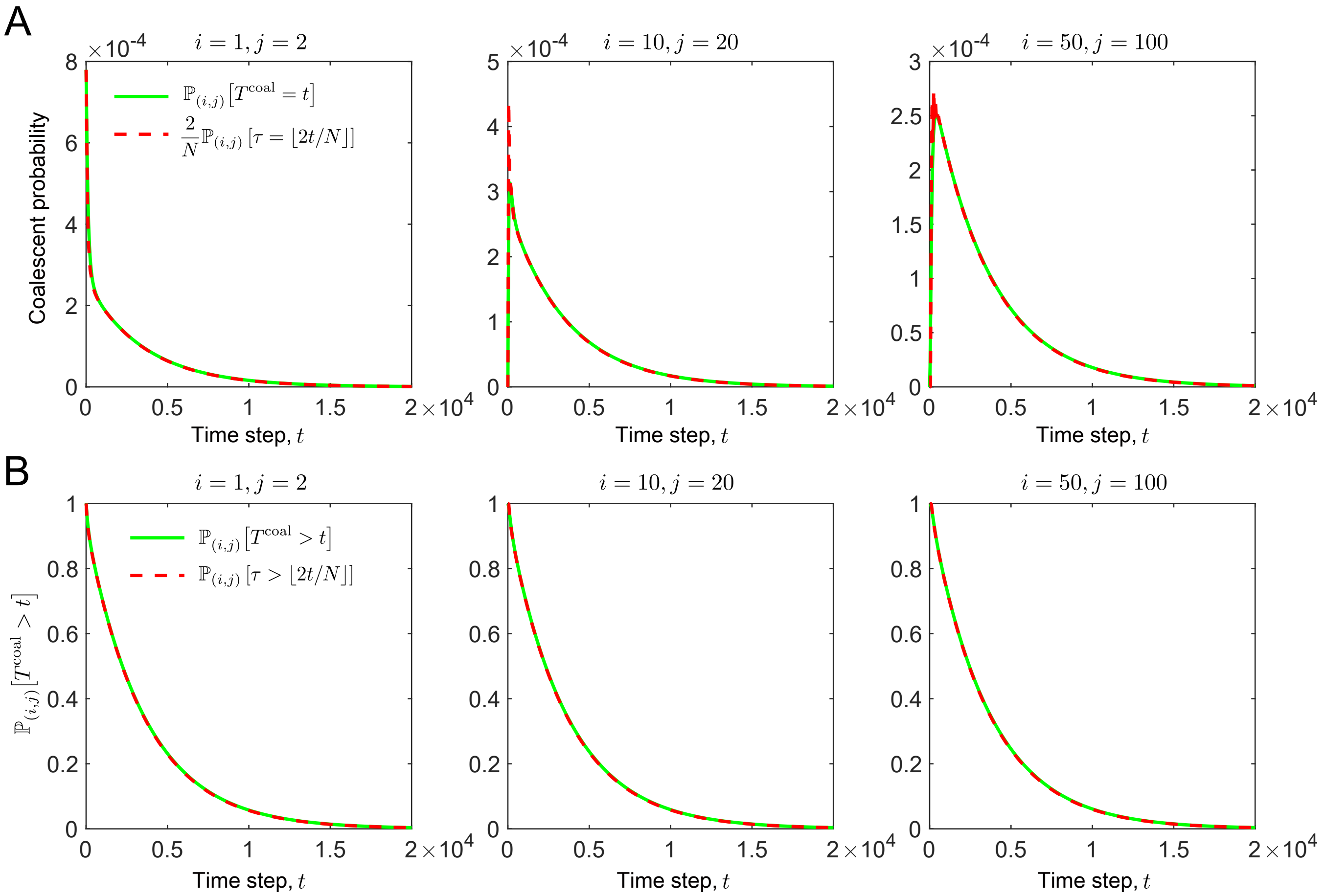

Fig. S15. Illustration of the coalescent probability over time. (*A*) We show the coalescent probability, when each individual has probability $1/N$ to be chosen to take a step (namely $\mathbb{P}_{(i,j)}\left[T^{\text{coal}} = t\right]$ in Eq. 19, green solid line) and when this probability is $1/2$ on each time step (namely $\mathbb{P}_{(i,j)}\left[\tau = t\right]$ in Eq. 21, red dashed line) on a scale-free network with 100 nodes. We show that $\mathbb{P}_{(i,j)}\left[T^{\text{coal}} = t\right]$ is approximately equal to $\frac{2}{N}\mathbb{P}_{(i,j)}\left[\tau = \lfloor 2t/N \rfloor\right]$ on three different pairs of nodes ($(1,2)$, $(10,20)$, and $(50,100)$). (*B*) We further present the accuracy of the approximation $\mathbb{P}_{(i,j)}\left[T^{\text{coal}} > t\right] \approx \mathbb{P}_{(i,j)}\left[\tau > \lfloor 2t/N \rfloor\right]$ over time on three different pairs of nodes. The scale-free network has an average degree of 6.

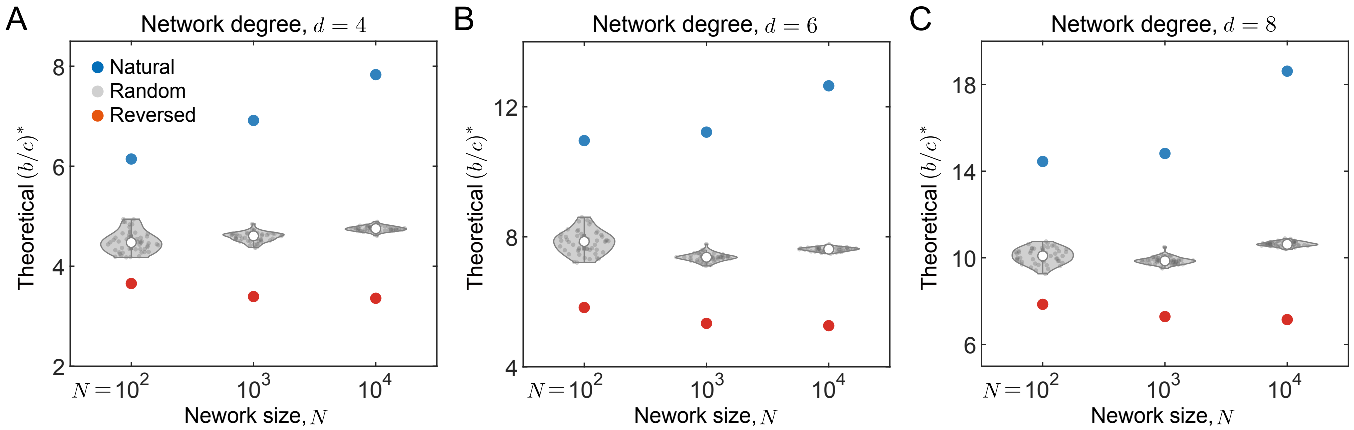

Fig. S16. Illustration of the critical ratio on large temporal networks We calculate the critical ratio, $(b/c)^*$, for temporal networks generated by preferential attachment at each time step. (*A-C*) For the network size $N = 10^2, 10^3, 10^4$, the results are consistent with temporal networks with $N = 20$ in Fig. S10, where the reversed ordering (orange) of a temporal network generated with preferential attachment has the lowest value of $(b/c)^*$, while the natural ordering (blue) hinders cooperation most. For each setting of network degree from $d = 4$ to $d = 8$, we show the values of 50 realizations of randomization of the ordering (grey), which lie between that of the natural and reversed orderings. Therefore, our conclusion of promoting the evolution of cooperation by reducing the priority of hubs can be confirmed on networks with different sizes and degrees.

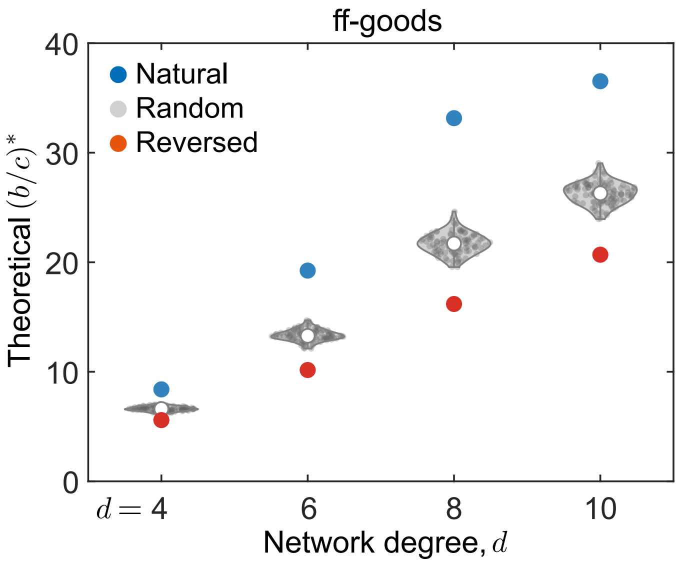

Fig. S17. Illustration of the critical ratio for different social goods. We calculate the critical ratio, $(b/c)^*$, with a fixed-cost, fixed-benefit model (10) at each step, where each cooperator divides a single benefit equally among all interaction partners, with a single cost paid. The result is consistent with death-Birth updating studied in the main text, where the reversed ordering (orange) of a temporal network generated with preferential attachment has the lowest value of $(b/c)^*$, while the natural ordering (blue) hinders cooperation most. For each setting of network degree from $d = 4$ to $d = 10$, we show the values of 100 realizations of randomization of the ordering (green), which lie between that of the natural and reversed orderings.

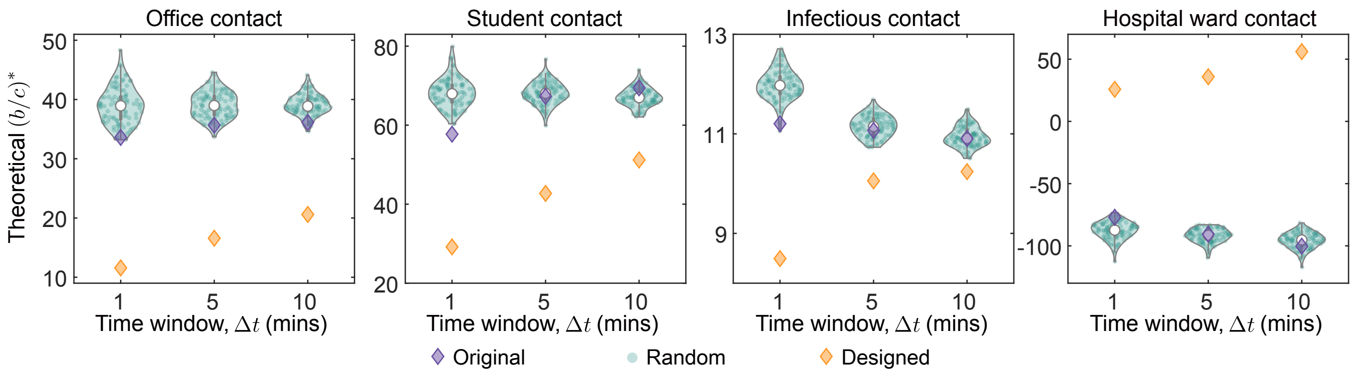

Fig. S18. Designing temporal interactions on empirical networks for imitation updating. We calculate the theoretical value of critical threshold $(b/c)^*$ under imitation updates on temporal interactions with both natural (purple diamond) and designed (golden diamond) ordering on four empirical datasets collected from from contacts in office (12), high school (13), exhibition (14), and hospital (15), respectively. As a control group, we plot the distribution of the random permuted snapshot sequence over 100 samples in cyan dots and shadows. For different values, Δt, our designed ordering of interactions facilitates the emergence of cooperation compared to both original and randomized interaction sequences.

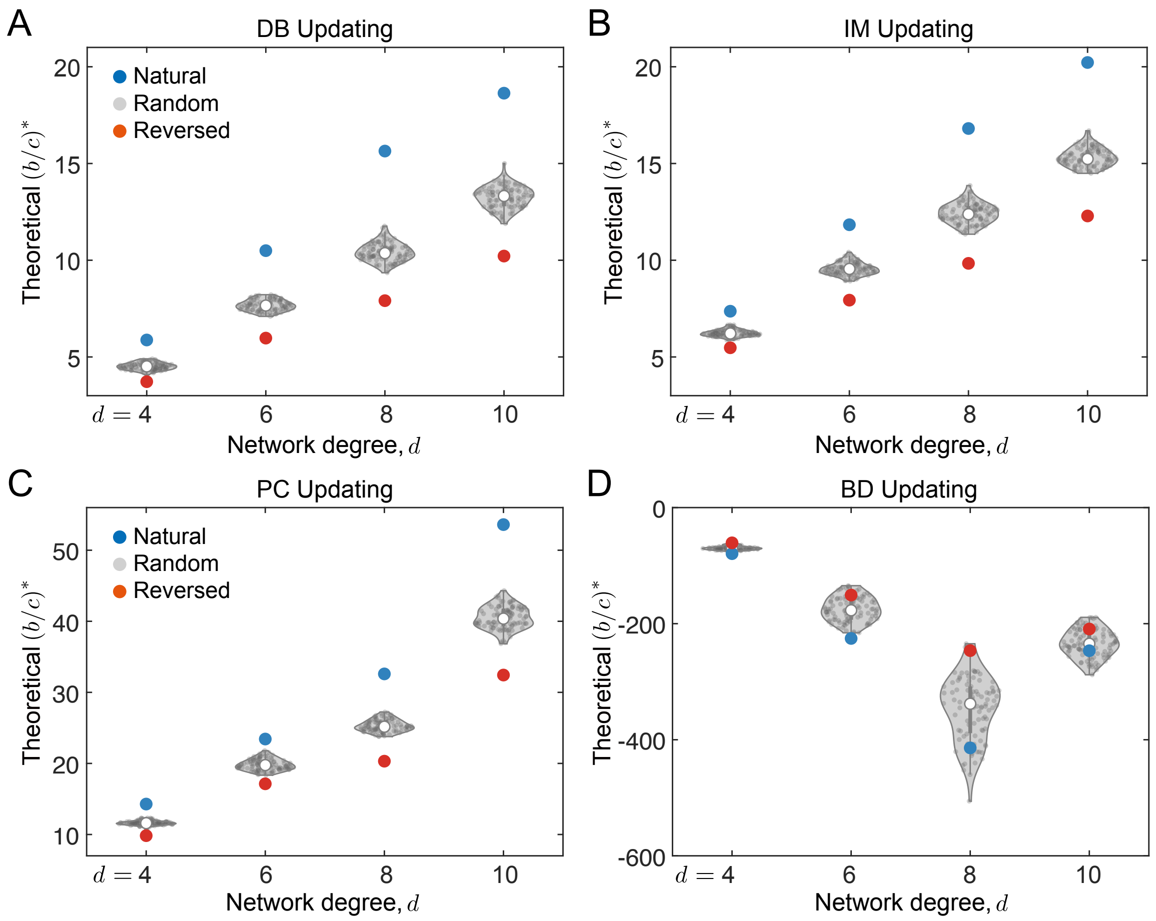

Fig. S19. Illustration of the critical ratio under different update rules. We calculate the critical ratio, $(b/c)^*$, for death-birth (DB), imitation (IM), pairwise-comparison (PC), and birth-death (BD) updating. (*A-C*) For IM and PC updating (10), the result is consistent with DB updating studied in the main text, where the reversed ordering (orange) of a temporal network generated with preferential attachment has the lowest value of $(b/c)^*$, while the natural ordering (blue) hinders cooperation most. For each setting of network degree from $d = 4$ to $d = 10$, we show the values of 100 realizations of randomization of the ordering (green), which lie between that of the natural and reversed orderings. (*D*) For the BD updating, competitions occur on cooperator-defector pairs, which brings disadvantages for the dispersal of cooperation and always leads to a negative $(b/c)^*$. Then the strategy of cooperation ($b > c > 0$) now transits to spite ($b < c < 0$), which is favored when $b/c < (b/c)^* < 0$ during the competition with defection. We show that the later presence of hubs (reversed ordering) can promote the evolution of spite over defection.

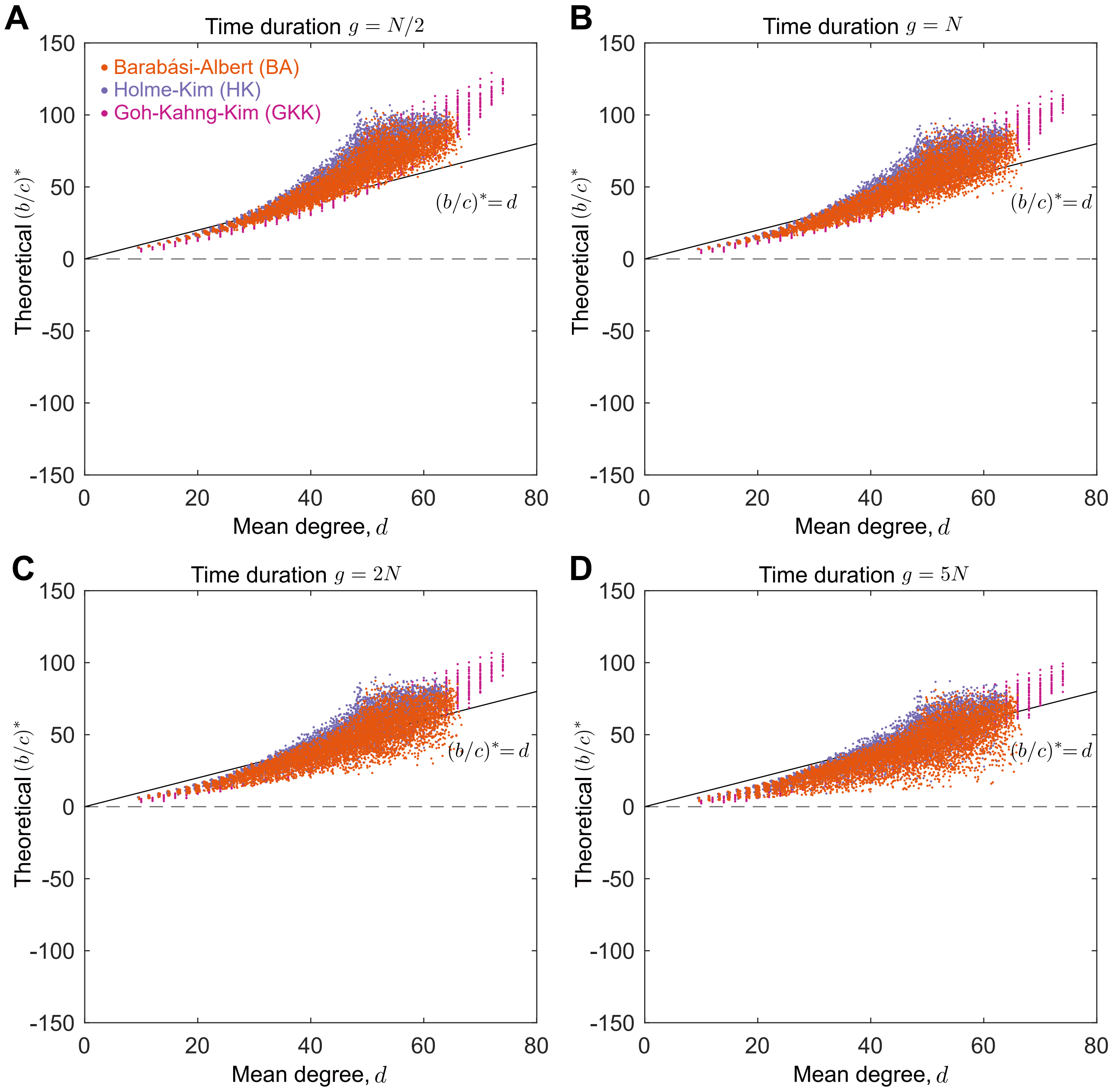

Fig. S20. Designing temporal interactions on heterogeneous replacement networks with different time duration. Here we consider different time duration $g = N/2, N, 2N, 5N$ on each snapshot, and calculate the critical ratio $(b/c)^{*}$ to confirm the effectiveness of our designed temporal interactions on heterogeneous replacement networks in (*A*)-(*D*), respectively. We show the scatter plot of critical ratio $(b/c)^{*}$ on Holme-Kim (HK) (17), Goh-Kahng-Kim (GKK) (18), and Barabási-Albert (BA) (16) scale-free networks. With our designed temporal interactions, all scale-free networks have positive values of $(b/c)^{*}$ with different g. Other parameters are the same as those of Fig. 4 in the main text.

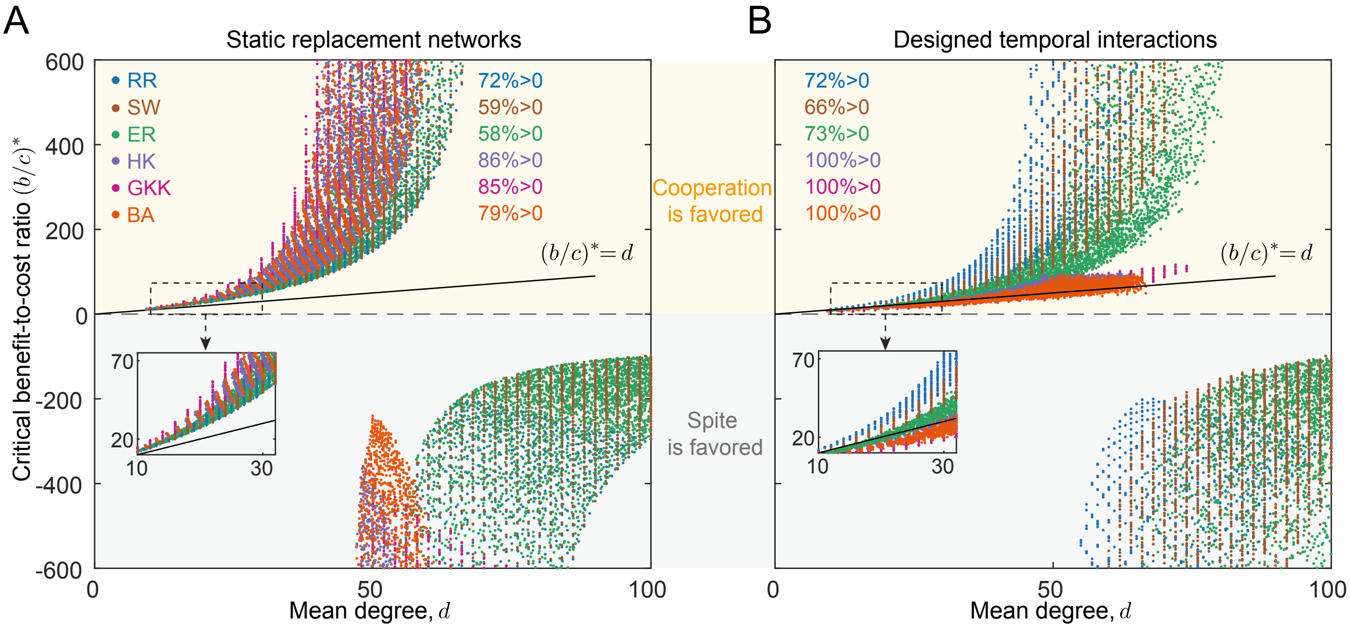

Fig. S21. Designing temporal interactions on various types of heterogeneous networks. (*A*) Scatter plot of the critical threshold, $(b/c)^*$, based on mean degree, d, on random regular (RR), small world (SW) (19), Erdös-Rényi (ER) (20), Holme-Kim (HK) (17), Goh-Kahng-Kim (GKK) (18), and Barabási-Albert (BA) (16) scale-free networks. Each type of network has negative value of $(b/c)^*$ when networks become dense, which implies that spite can be favored and there is no possibility for those networks to favor cooperation. (*B*) With our designed temporal interactions, all scale-free networks have positive values of $(b/c)^*$, which is approximately equal to the average degree (d) of the network. In contrast, the proportion with a positive critical threshold on random regular networks remains unchanged. Other parameters are the same as those of Fig. 4 in the main text.

References

1. B Allen, et al., Evolutionary dynamics on any population structure. *Nature* **544**, 227–230 (2017).
2. K Sigmund, *The Calculus of Selfishness*. (Princeton University Press), (2010).
3. MA Nowak, A Sasaki, C Taylor, D Fudenberg, Emergence of cooperation and evolutionary stability in finite populations. *Nature* **428**, 646–650 (2004).
4. B Allen, A McAvoy, A mathematical formalism for natural selection with arbitrary spatial and genetic structure. *J. Math. Biol.* **78**, 1147–1210 (2019).
5. A McAvoy, B Allen, Fixation probabilities in evolutionary dynamics under weak selection. *J. Math. Biol.* **82**, 1–41 (2021).
6. RA Fisher, *The Genetical Theory of Natural Selection*. (Clarendon Press), (1930).
7. PD Taylor, Allele-Frequency Change in a Class-Structured Population. *Am. Nat.* **135**, 95–106 (1990).
8. W Maciejewski, F Fu, C Hauert, Evolutionary game dynamics in populations with heterogenous structures. *PLoS Comput. Biol.* **10**, e1003567 (2014).
9. Y Meng, SP Cornelius, YY Liu, A Li, Dynamics of collective cooperation under personalised strategy updates. *Nat. Commun.* **15**, 3125 (2024).
10. A McAvoy, B Allen, MA Nowak, Social goods dilemmas in heterogeneous societies. *Nat. Hum. Behav.* **4**, 819–831 (2020).
11. JT Cox, Coalescing random walks and voter model consensus times on the torus in Z^d. *Ann. Probab.* **17**, 1333–1366 (1989).
12. M Génois, et al., Data on face-to-face contacts in an office building suggest a low-cost vaccination strategy based on community linkers. *Netw. Sci.* **3**, 326–347 (2015).
13. J Fournet, A Barrat, Contact patterns among high school students. *PLoS One* **9**, e107878 (2014).
14. L Isella, et al., What's in a crowd? analysis of face-to-face behavioral networks. *J. Theor. Biol.* **271**, 166–180 (2011).
15. P Vanhems, et al., Estimating potential infection transmission routes in hospital wards using wearable proximity sensors. *PLoS One* **8**, e73970 (2013).
16. AL Barabási, R Albert, Emergence of scaling in random networks. *Science* **286**, 509–512 (1999).
17. P Holme, BJ Kim, Growing scale-free networks with tunable clustering. *Phys. Rev. E* **65**, 026107 (2002).
18. KI Goh, B Kahng, D Kim, Universal behavior of load distribution in scale-free networks. *Phys. Rev. Lett.* **87**, 278701 (2001).
19. DJ Watts, SH Strogatz, Collective dynamics of 'small-world' networks. *Nature* **393**, 440–442 (1998).
20. P Erdös, A Rényi, On random graphs I. *Publ. Math. (Debrecen)* **6**, 290–297 (1959).
